# Homogeneity Test of the First-Order Agreement Coefficient in a Stratified Design

**DOI:** 10.3390/e25030536

**Published:** 2023-03-20

**Authors:** Mingrui Xu, Zhiming Li, Keyi Mou, Kalakani Mohammad Shuaib

**Affiliations:** College of Mathematics and System Science, Xinjiang University, Urumqi 830046, China

**Keywords:** Gwet’s AC_1_, homogeneity test, asymptotic statistic, exact method

## Abstract

Gwet’s first-order agreement coefficient (AC${}_{1}$) is widely used to assess the agreement between raters. This paper proposes several asymptotic statistics for a homogeneity test of stratified AC${}_{1}$ in large sample sizes. These statistics may have unsatisfactory performance, especially for small samples and a high value of AC${}_{1}$. Furthermore, we propose three exact methods for small pieces. A likelihood ratio statistic is recommended in large sample sizes based on the numerical results. The exact E approaches under likelihood ratio and score statistics are more robust in the case of small sample scenarios. Moreover, the exact E method is effective to a high value of AC${}_{1}$. We apply two real examples to illustrate the proposed methods.

## 1. Introduction

In the medical field, it is necessary to judge the accuracy and the interchangeability of different diagnostics. Inter-rater agreement is widely used to quantify the closeness of ratings for subjects by two raters. The recommendation of an efficient and economical method should guarantee a high degree of agreement between its result and the gold-standard method. A simple example is that independent raters A and B assess each subject with binary outcomes (e.g., +/−, and Yes/No). Let $n_{ij}\left(i,j=1,2\right)$ be the numbers of independent subjects judged by two raters as (+,+), (−,+), (+,−), and (−,−), and $P_{ij}\left(i,j=1,2\right)$ be the corresponding probabilities, respectively. Denote $n=n_{1+}+n_{2+}=n_{+1}+n_{+2}=\sum_{i=1}^{2}\sum_{j=1}^{2}n_{ij}$. The data can be arranged into a 2×2 original table (Table 1).

Researchers have developed several indices by which to measure the degree of agreement between raters on a nominal scale category, where the unordered categories are independent, mutually exclusive, and exhaustive. Denote $P_{i}=Pr\left(the\;probability\;is\;classified\;as\;“+”\;by\;Rater\;i\right)$, i=A,B. We call $P_{i}\left(i=A,B\right)$ as the marginal probability. Cohen [1] showed that the $\chi^{2}$ test was indefensible because of the null hypothesis with independence, not agreement. Furthermore, he presented the kappa coefficient to compute the extent of agreement between raters. For the problem of nominal scale agreement between raters A and B in Table 1, there are only two relevant quantities: the overall agreement probability $p_{a}$, and the chance–agreement probability $p_{c}$. Cohen’s kappa coefficient is defined by $\kappa =\left(p_{a}-p_{c}\right)/\left(1-p_{c}\right),$ where $p_{a}=P_{11}+P_{22}$, $p_{c}=P_{A}P_{B}+\left(1-P_{A}\right)\left(1-P_{B}\right)$. That is to say, the coefficient κ is the proportion of agreement after the removal of chance agreement. Suppose that the distribution of proportions over the categories for the population is known and is taken to be equal for the judges. Thus, Scott [2] proposed π coefficient $\pi =\left(p_{a}-p_{\pi }\right)/\left(1-p_{\pi }\right)$, where $p_{\pi }$ is the percent agreement to be expected on the basis of chance, and $p_{\pi }={\left(\frac{P_{A}+P_{B}}{2}\right)}^{2}+{\left(1-\frac{P_{A}+P_{B}}{2}\right)}^{2}$. Despite the wide range of applications, the limitations of these coefficients have two main aspects: (i) it highly depends on marginal probabilities [3], and (ii) it is often affected by the composition of the population for subjects easy or difficult to agree upon [4]. For example, Cicchetti and Feinstein [3] illustrated one of the limitations by an example: $n_{11}=118$, $n_{12}=5$, $n_{21}=2$, and $n_{22}=0$. Through simple calculation, $p_{a}=118/125+0/125=0.944$, $p_{c}=\left(123/125\right)\times \left(120/125\right)+\left(5/125\right)\times \left(2/125\right)=0.9453$. Thus, the estimator $\hat{\kappa }=\left(0.944-0.9456\right)/\left(1-0.9456\right)=-0.0234<0$. For Scott’s π coefficient, we have $\hat{\pi }=-0.0288$ because $p_{\pi }=0.9456$. It is unreasonable that a high agreement has low κ and π coefficients. To solve the problem, some alternative indices have been derived to measure the consistency, such as Holley and Guilford’s G index [5], Aickin’s α agreement parameter [6], Andr$\overset{´}{e}$s and Marzo’s delta measure [7]. Gwet [8] revealed the origin of these limitations and proposed the first-order agreement coefficient (AC${}_{1}$) as an alternative index. The definition of this coefficient is based on two premises: (a) chance agreement occurs when at least one rater rates an individual randomly, and (b) only an unknown portion of observed ratings is subjected to randomness. Define two events

$$\begin{array}{c} G=\{both\;raters\;agree\},\;\;\;R=\{at\;least\;one\;rater\;performs\;random\;rating\}. \end{array}$$

Thus, the probability of agreement expected by chance can be defined by $p_{e}≜P\left(G\cap R\right)=P\left(R\right)P\left(G|R\right).$ Generally, a random rating may classify an individual into either category with the same probability of 1/2. Since agreement may occur in either type, we have $P\left(G|R\right)=2\times {\left(1/2\right)}^{2}=1/2$. As for the probability of random rating P(R), a normalized measure of randomness (Ψ) is used to approximate it as follows,

$$P\left(R\right)\approx \Psi =\frac{\pi_{+}\left(1-\pi_{+}\right)}{1/2(1-1/2)}=4\pi_{+}\left(1-\pi_{+}\right),$$

where $\pi_{+}$ represents the probability that a random rater classifies a randomly chosen individual into the “+” category. That is to say, $p_{e}$ can be quantified by $p_{e}^{*}=P\left(G|R\right)\Psi =2\pi_{+}\left(1-\pi_{+}\right)$. Then, the AC${}_{1}$ coefficient can be expressed as $\gamma =\left(p_{a}-p_{e}^{*}\right)/\left(1-p_{e}^{*}\right),$ where $p_{a}$ denotes the agreement probability. In the above example, ${\hat{\pi }}_{+}=\left(123/125+120/125\right)/2=0.9720$ and $p_{e}^{*}=0.0544$. By the definition of AC${}_{1}$, we have γ=0.9408. Thus, the AC${}_{1}$ coefficient is more consistent with the observed extent of agreement than Cohen’s κ and Scott’s π coefficients. There have been quite a few pieces of literature about agreement coefficients [9,10,11].

As with Scott’s π coefficient, Ohyama [12] assumed that two raters have a common marginal probability, that is, $P_{A}=P_{B}≜\pi_{+}$. Thus, $P_{12}=P_{21}$, and Table 1 can be simplified as Table 2. Define

$$\begin{array}{c} X_{ij}=\left\{\begin{array}{cc} 1, & if\;rater\;i\;classifies\;the\;subject\;j\;into\;category\;+,\\ 0, & otherwise \end{array}\right. \end{array}$$

for i=A,B,j=1,2,…,n. Suppose that the underlying probability of classifying a subject depends not on raters but subjects, which is $P\left(X_{ij}=1|j\right)=p_{j}$. We can obtain the overall agreement probability $\left(p_{a}\right)$ based on the idea of Vanbelle and Albert [13]. The agreement can occur in “(+,+)” and “(−,−)”, and the corresponding probabilities for *j*th subject are $P_{11j}=p_{j}^{2}$ and $P_{22j}={\left(1-p_{j}\right)}^{2}$, respectively. Thus, the agreement probability of two raters for the *j*th subject is $p_{aj}=p_{j}^{2}+{\left(1-p_{j}\right)}^{2}$. We denote the mean of positive classification probability as $E\left(p_{j}\right)=\sum_{j=1}^{n}p_{j}/n≜\pi_{+}$, and the corresponding variance as $\mathrm{Var}\left(p_{j}\right)=\sum_{j=1}^{n}{\left(p_{j}-\pi_{+}\right)}^{2}/n≜\sigma^{2}$, where *n* is the size of the population. Then, the probabilities of “(+,+)” and “(−,−)” ratings over the population are $P_{11}=E\left(p_{j}^{2}\right)=Var\left(p_{j}\right)+{\left(E\left(p_{j}\right)\right)}^{2}=\sigma^{2}+\pi_{+}^{2}$ and $P_{22}=E\left({\left(1-p_{j}\right)}^{2}\right)=1-2\pi_{+}+\sigma^{2}+\pi_{+}^{2}$. Finally, the agreement probability over the population is $p_{a}=P_{11}+P_{22}=1+2\left(\sigma^{2}-\pi_{+}\left(1-\pi_{+}\right)\right)$. The AC${}_{1}$ coefficient (γ) for a binary outcome judged by two raters is rewritten by

(1) $$\begin{array}{c} \gamma =\frac{p_{a}-p_{e}^{*}}{1-p_{e}^{*}}=\frac{1+2\sigma^{2}-4\pi_{+}\left(1-\pi_{+}\right)}{1-2\pi_{+}\left(1-\pi_{+}\right)}. \end{array}$$

Up until now, the application [14,15] and the statistical inference [12] of the AC${}_{1}$ have been concentrated at the situation without stratification. However, the ignorance of confounding variables or covariates may lead to a biased conclusion. Researchers often stratify the data into multiple strata to control the influence of these factors. A stratified analysis is applied to evaluate the relationship between the nontreatment factors of a clinical trial (age, gender, or severity of disease, etc.) and agreement. A test of homogeneity is the first step of the stratified analysis. It is essential to analyze the factors that lead to heterogeneity when we reject the homogeneity hypothesis. Suppose *K* levels of the subject covariates are introduced into Table 2 for two raters with binary outcome, and the data can be arranged in a 3×K table of observed cell counts. Generally speaking, a sample can be classified as a large or small sample by the sample size. Hannah et al. [16] analyzed the data about the alcohol-drinking status of twins. A subject is categorised as nondrinker if he/she consumes less than 30 gm alcohol per week, and otherwise is a drinker. Thus, the binary outcome is the drinking status (drinker or nondrinker). A number of same-sex twins are stratified by zygosity, including monozygotic (MZ), and dizygotic (DZ). Nam [17] used the kappa index to investigate the agreement of alcohol-drinking status between twins. The data structure of male twins is shown in Table 3. The large-sample inference has been performed for the data type, including score, likelihood ratio, and Wald-type statistics [18]. Honda and Ohyama [19] proposed score and goodness-of-fit tests for the homogeneity test of stratified AC${}_{1}$. Unfortunately, both tests performed poorly due to the conservative or liberal type I error rates, especially for small sample sizes. Meanwhile, a high AC${}_{1}$ may lead to conservative type I error rates for small and moderate sample sizes.

In practice, we often encounter small sample cases of agreement data, for example, a clinical trial about coronavirus disease 2019 (COVID-19) [20]. In this trial, the enzyme-linked immunosorbent assay (ELISA) and gold-standard methods are used to detect the novel coronavirus IgG and IgM antibodies, classifying each of them as either positive (+) or negative (−). ELISA positive criterion is that the sample’s optical density (OD) value is greater than or equal to the critical value. The positive criterion of the gold-standard method is the appearance of two colored bands. Table 4 lists the data stratified by the IgG and the IgM antibodies (K=2), 17 patients in each group. Similar to Table 3, “One” entry corresponds to the number of “(+,−)” and “(−,+)”.

Unfortunately, asymptotic test statistics do not apply to small data. Exact approaches are effective for small samples, such as Fisher’s exact test [21,22,23], and its extensions [24,25,26]. A conservative performance of Fisher’s exact method supported the appearance of other exact approaches. We note that there exist nuisance parameters in the model of AC${}_{1}$ coefficient. Significant progress has been achieved in the elimination of nuisance parameters for decades [27,28,29,30,31]. By fixing the marginal totals in the contingency table, Mehta [27] extensively used the conditional test (referred to as the C approach) to analyze various classical categorical data. Liddell [28] derived a test based on the exact distribution of the difference in sample proportions. As an alternative, Storer and Kim [29] modified Liddell’s exact test, abbreviated as the E approach. Basu [30] provided a new procedure by maximizing the tail probability over the whole range of parameters, called the M approach. The global maximum is a challenge when the parameter space is not finite. Lloyd [31] pointed out the weakness of the M approach, and he suggested a so-called E+M approach by defining the tail area with the E approach and maximizing the tail probability over the parameter space. Generally, E, M, and E+M approaches are called unconditional tests. Tang et al. [32] showed that the exact conditional approach was generally inferior to the exact unconditional approach for small samples. Shan and Wilding [33] compared asymptotic and exact procedures for the kappa coefficient in a 2×2 table. However, little work has been carried out in extending the exact approaches to test the homogeneity of the AC${}_{1}$ coefficients across several independent strata.

This paper aims to propose asymptotic and exact methods for the homogeneity test of stratified AC${}_{1}$. The novelty and contribution are shown by three main aspects as follows. (i) For large sample sizes, we propose two asymptotic statistics, including likelihood ratio and Wald-type tests, to extend the study of homogeneity test in Honda and Ohyama [19] under large sample sizes. Our results show that the likelihood ratio test is more robust than other tests regarding type I error rates. The powers of these tests are close to each other. Thus, we recommend the likelihood ratio test for large samples’ homogeneity test of stratified AC${}_{1}$. (ii) Based on the asymptotic statistics, we derive three exact approaches (E, M, and E+M methods) to investigate the small sample cases (n=10,25). These exact methods can effectively improve the performance of the homogeneity test concerning type I error rates. Among these methods, the exact E approaches based on likelihood ratio and score tests are more robust in small samples. (iii) We investigate the strengths and weaknesses of asymptotic and exact methods through plentiful numerical analyses, respectively. Some beneficial conclusions are obtained from the analyses of actual examples. The rest of this paper is organized as follows. In Section 2, we review the AC${}_{1}$ coefficient in a stratified condition and establish a probability model. The maximum likelihood method and iterative algorithm are used to estimate the unknown parameters. We further review the score statistic and derive two asymptotic test statistics for large samples in Section 3. Based on these statistics, several exact methods are used for small sample sizes in Section 4. In Section 5, we conduct numerical studies to investigate the performance of all the derived methods regarding type I error rates and powers. In Section 6, we study the aforementioned real examples of large and small samples to illustrate these methods. Finally, a brief conclusion is given in Section 7.

## 2. A Probability Model and Homogeneity Test

Following Ohyama [12], we introduce *K* covariates into Table 2 and establish a probability model. Suppose that *N* subjects are divided into *K* independent strata. In the *k*th (k=1,2,…,K) stratum, there are $n_{1k},n_{2k}$, and $n_{3k}$ subjects in the three categories. Denote $n_{k}=\sum_{l=1}^{3}n_{lk}$ as the total number of subjects in the *k*th stratum. Table 5 shows the data structure across the strata.

For the stratified analysis, we need to construct AC${}_{1}$ for each stratum. Let $X_{kij}$ be an indicator of the *i*th (i=1,2) rater’s judgement for the *j*th $\left(j=1,2,\ldots ,n_{K}\right)$ subject in the *k*th (k=1,2,…,K) stratum. If there is a positive “(+)” classification, then $X_{kij}=1$, and otherwise 0. Ohyama [12] assumed that the underlying probability of classifying a subject does not depend on raters but on subjects; that is, $Pr\left(X_{kij}=1|j\right)=p_{kj}$. The *N* subjects are classified into *K* strata based on covariates, and every stratum has different subjects. Thus, the data of every stratum is independent of each other. Denote $E\left(p_{kj}\right)=\sum_{j=1}^{n_{k}}p_{kj}/n_{k}≜\pi_{k}$, and $Var\left(p_{kj}\right)=\sum_{j=1}^{n_{k}}{\left(p_{kj}-\pi_{k}\right)}^{2}/n_{k}≜\sigma_{k}^{2}$. Then, AC${}_{1}$ of the *k*th stratum is

$$\gamma_{k}=\frac{1+2[\sigma_{k}^{2}-2\pi_{k}\left(1-\pi_{k}\right)]}{1-2\pi_{k}\left(1-\pi_{k}\right)},\;\;k=1,2,\ldots ,K.$$

Suppose that $P_{1k}\left(\gamma_{k},\pi_{k}\right)$, $P_{2k}\left(\gamma_{k},\pi_{k}\right)$, and $P_{3k}\left(\gamma_{k},\pi_{k}\right)$ are the corresponding probabilities in the *k*th stratum, where $\pi_{k}$ and $\gamma_{k}$ are the common positive classification probability and the AC${}_{1}$ coefficient, respectively. As the AC${}_{1}$ coefficient in the *k*th stratum, $\gamma_{k}$ includes the information of $\pi_{k}$ and $\sigma_{k}$. It is obvious that there is no one-to-one correspondence between $\gamma_{k}$ and $\pi_{k}$. Denote $n_{k}={\left(n_{1k},n_{2k},n_{3k}\right)}^{T}$, and $P_{k}=(P_{1k}\left(\gamma_{k},\pi_{k}\right)$, $P_{2k}\left(\gamma_{k},\pi_{k}\right)$, $P_{3k}\left(\gamma_{k},\pi_{k}\right){)}^{T}$. For the *k*th stratum, $n_{k}\left(k=1,2,\ldots ,K\right)$ follows a trinomial distribution. Thus, the probability density of $n_{k}$ is expressed as follows:

(2) $$\begin{array}{c} f\left(P_{k}|n_{k}\right)=\frac{n_{k}!}{n_{1k}!n_{2k}!n_{3k}!}P_{1k}^{n_{1k}}P_{2k}^{n_{2k}}P_{3k}^{n_{3k}}. \end{array}$$

**Figure 1:**
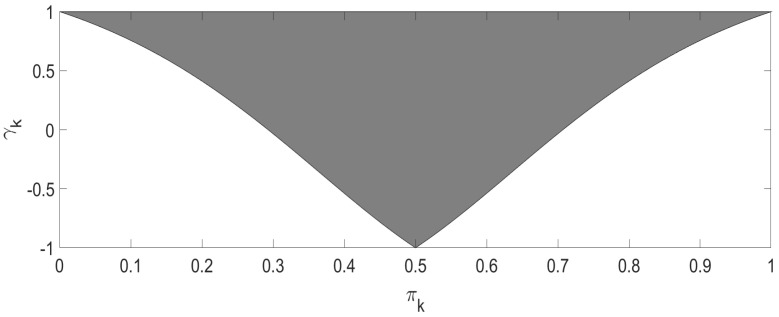
The range of $\gamma_{k}$.

Through calculation, the probabilities $P_{lk}\left(l=1,2,3,k=1,2,\ldots ,K\right)$ are obtained by

(3) $$\begin{array}{c} \left\{\begin{array}{c} P_{1k}\left(\gamma_{k},\pi_{k}\right)=\pi_{k}\left(2-\pi_{k}\right)-1/2+\gamma_{k}\left(1-2\pi_{k}\left(1-\pi_{k}\right)\right)/2,\\ P_{2k}\left(\gamma_{k},\pi_{k}\right)=\left(1-2\pi_{k}\left(1-\pi_{k}\right)\right)\left(1-\gamma_{k}\right),\\ P_{3k}\left(\gamma_{k},\pi_{k}\right)=\left(1-\pi_{k}\right)\left(1+\pi_{k}\right)-1/2+\gamma_{k}\left(1-2\pi_{k}\left(1-\pi_{k}\right)\right)/2, \end{array}\right. \end{array}$$

where $0\leq P_{ik}\left(\gamma_{k},\pi_{k}\right)\leq 1$ and $\sum_{i=1}^{3}P_{ik}\left(\gamma_{k},\pi_{k}\right)=1$. Figure 1 shows the admissible range of $\gamma_{k}$, satisfying

$$\frac{2-(1-|1-2\pi_{k}|)(3+|1-2\pi_{k}\left|\right)}{2-(1-|1-2\pi_{k}|)(1+|1-2\pi_{k}\left|\right)}\leq \gamma_{k}\leq 1.$$

Our work is interested in testing whether the AC${}_{1}$ coefficients $\gamma_{k}\left(k=1,2,\ldots ,K\right)$ are homogeneous among the *K* independent strata, that is,

$$\begin{array}{c} H_{0}:\gamma_{1}=\cdots =\gamma_{K}≜\gamma \;\;\mathrm{vs}\;\;H_{a}:\gamma_{k}\;\mathrm{is}\;\mathrm{not}\;\mathrm{all}\;\mathrm{the}\;\mathrm{same}, \end{array}$$

Denote $\pi =(\pi_{1},\ldots ,\pi_{K})$ and $\gamma =(\gamma_{1},\ldots ,\gamma_{K})$. First, we calculate the unknown parameters under the alternative hypothesis $H_{a}$. The corresponding log-likelihood function of the observed data $N=(n_{1},n_{2},\ldots ,n_{K})$ is

$$\begin{array}{c} \begin{array}{cc} l(\gamma ,\pi |N) & =\log\left(\prod_{k=1}^{K}f\left(P_{k}|n_{k}\right)\right)=\log\left(\prod_{k=1}^{K}\frac{n_{k}!}{n_{1k}!n_{2k}!n_{3k}!}P_{1k}^{n_{1k}}P_{2k}^{n_{2k}}P_{3k}^{n_{3k}}\right)\\ & =\sum_{k=1}^{K}\left[n_{1k}\log P_{1k}\left(\gamma_{k},\pi_{k}\right)+n_{2k}\log P_{2k}\left(\gamma_{k},\pi_{k}\right)+n_{3k}\log P_{3k}\left(\gamma_{k},\pi_{k}\right)\right]+C\\ & ≜\sum_{k=1}^{K}l_{k}\left(\gamma_{k},\pi_{k}|n_{k}\right)+C, \end{array} \end{array}$$

where $C=\log(\prod_{k=1}^{K}\frac{n_{k}!}{n_{1k}!n_{2k}!n_{3k}!})$ is a constant, and $l_{k}\left(\gamma_{k},\pi_{k}|n_{k}\right)$ is the log-likelihood function of the *k*th stratum under $H_{a}$. Let ${\hat{\gamma }}_{k}$ and ${\hat{\pi }}_{k}\;\left(k=1,2,\ldots ,K\right)$ be the unconstrained maximum likelihood estimates (MLEs) of $\gamma_{k}$ and $\pi_{k}$ under $H_{a}$. By solving the following equations,

$$\begin{array}{c} \begin{array}{cc} \frac{\partial l_{k}}{\partial \pi_{k}} & =\frac{2n_{2k}\left(2\pi_{k}-1\right)}{2\pi_{k}^{2}-2\pi_{k}+1}-\frac{2n_{3k}\left(\gamma_{k}+2\pi_{k}-2\pi_{k}\gamma_{k}\right)}{1-2\pi_{k}^{2}+\gamma_{k}\left(2\pi_{k}^{2}-2\pi_{k}+1\right)}+\frac{2n_{1k}\left(2-2\pi_{k}-\gamma_{k}+2\pi_{k}\gamma_{k}\right)}{4\pi_{k}+\gamma_{k}\left(2\pi_{k}^{2}-2\pi_{k}+1\right)-2\pi_{k}^{2}-1}=0,\\ \frac{\partial l_{k}}{\partial \gamma_{k}} & =\frac{n_{2k}}{\gamma_{k}-1}+\frac{n_{1k}\left(2\pi_{k}^{2}-2\pi_{k}+1\right)}{4\pi_{k}+\gamma_{k}\left(2\pi_{k}^{2}-2\pi_{k}+1\right)-2\pi_{k}^{2}-1}+\frac{n_{3k}\left(2\pi_{k}^{2}-2\pi_{k}+1\right)}{1-2\pi_{k}^{2}+\gamma_{k}\left(2\pi_{k}^{2}-2\pi_{k}+1\right)}=0, \end{array} \end{array}$$

we have

$${\hat{\pi }}_{k}=\frac{2n_{1k}+n_{2k}}{2n_{k}},\;{\hat{\gamma }}_{k}=1-\frac{2n_{k}n_{2k}}{n_{k}^{2}+{\left(n_{1k}-n_{3k}\right)}^{2}},\;k=1,2,\ldots ,K.$$

Next, we estimate the parameters γ and $\pi =(\pi_{1},\ldots ,\pi_{K})$ under the null hypothesis $H_{0}:\gamma_{1}=\gamma_{2}=\cdots =\gamma_{K}≜\gamma$. The log-likelihood function is rewritten by

$$\begin{array}{c} \begin{array}{cc} l_{0}\left(\gamma ,\pi |N\right) & =\sum_{k=1}^{K}\{n_{1k}\log\left[\pi_{k}\left(2-\pi_{k}\right)-\frac{1}{2}+\frac{\gamma }{2}\left(1-2\pi_{k}\left(1-\pi_{k}\right)\right)\right]+n_{2k}\log\left[\left(1-2\pi_{k}\left(1-\pi_{k}\right)\right)\left(1-\gamma \right)\right]\\ & \;\;\;\;+n_{3k}\log\left[\left(1-\pi_{k}\right)\left(1+\pi_{k}\right)-\frac{1}{2}+\frac{\gamma }{2}\left(1-2\pi_{k}\left(1-\pi_{k}\right)\right)\right]\}+C\\ & ≜\sum_{k=1}^{K}l_{0k}\left(\gamma ,\pi_{k}|n_{k}\right)+C, \end{array} \end{array}$$

where $l_{0k}\left(\gamma ,\pi_{k}|n_{k}\right)$ is the log-likelihood function of the *k*th stratum under $H_{0}$. Let $\tilde{\gamma }$ and ${\tilde{\pi }}_{k}\;\left(k=1,2,\ldots ,K\right)$ be the constrained MLEs of γ and $\pi_{k}$ under $H_{0}$. Similarly, we can differentiate $l_{0}\left(\gamma ,\pi |N\right)$ to γ and $\pi_{k}$, and set them to zero as follows:

$$\begin{array}{c} \begin{array}{cc} \frac{\partial l_{0}}{\partial \pi_{k}}= & \frac{2n_{2k}\left(2\pi_{k}-1\right)}{2\pi_{k}^{2}-2\pi_{k}+1}-\frac{2n_{3k}\left(\gamma +2\pi_{k}-2\pi_{k}\gamma \right)}{1-2\pi_{k}^{2}+\gamma \left(2\pi_{k}^{2}-2\pi_{k}+1\right)}+\frac{2n_{1k}\left(2-2\pi_{k}-\gamma +2\pi_{k}\gamma \right)}{4\pi_{k}+\gamma \left(2\pi_{k}^{2}-2\pi_{k}+1\right)-2\pi_{k}^{2}-1}=0,\\ \frac{\partial l_{0}}{\partial \gamma }= & \sum_{k=1}^{K}\left[\frac{n_{2k}}{\gamma -1}+\frac{n_{1k}\left(2\pi_{k}^{2}-2\pi_{k}+1\right)}{4\pi_{k}+\gamma \left(2\pi_{k}^{2}-2\pi_{k}+1\right)-2\pi_{k}^{2}-1}+\frac{n_{3k}\left(2\pi_{k}^{2}-2\pi_{k}+1\right)}{1-2\pi_{k}^{2}+\gamma \left(2\pi_{k}^{2}-2\pi_{k}+1\right)}\right]=0. \end{array} \end{array}$$

However, there are no closed-form solutions for the above equations. The Fisher scoring algorithm is used to obtain the constrained MLEs. Three steps describe the iteration process as follows.

(i) Given the initial values $\gamma^{\left(0\right)}=0.5$, and $\pi_{k}^{\left(0\right)}=\left(2n_{1k}+n_{2k}\right)/\left(2n_{k}\right)$ in the *k*th stratum.
(ii) The (t+1)-th approximates of γ and π can be updated by
  $$\begin{array}{c} \left[\begin{array}{c} \;\gamma^{\left(t+1\right)}\\ \pi^{\left(t+1\right)} \end{array}\right]=\left[\begin{array}{c} \;\gamma^{\left(t\right)}\\ \pi^{\left(t\right)} \end{array}\right]+I_{1}^{-1}\left(\gamma ,\pi \right)\times \left[\begin{array}{c} \;\frac{\partial l}{\partial \gamma }\\ \frac{\partial l}{\partial \pi } \end{array}\right]{|}_{\gamma =\gamma^{\left(t\right)},\pi =\pi^{\left(t\right)}}, \end{array}$$
  where $\pi ={\left(\pi_{1},\ldots ,\pi_{K}\right)}^{T}$, $\frac{\partial l}{\partial \pi }={\left(\frac{\partial l}{\partial \pi_{1}},\ldots ,\frac{\partial l}{\partial \pi_{K}}\right)}^{T}$, and $I_{1}$ is the (K+1)×(K+1) Fisher information matrix (Appendix A.1).
(iii) Repeat the processes (i)–(ii) until the results converge.

## 3. Asymptotic Methods

### 3.1. Likelihood Ratio Statistic $T_{L}$

The unconstrained and constrained MLEs construct a likelihood ratio test statistic. It is defined by

$$T_{L}=2\left[l\left(\hat{\gamma },\hat{\pi }|N\right)-l_{0}\left(\tilde{\gamma },\tilde{\pi }|N\right)\right]=2\sum_{k=1}^{K}\left[l_{k}\left({\hat{\gamma }}_{k},{\hat{\pi }}_{k}|n_{k}\right)-l_{0k}\left(\tilde{\gamma },{\tilde{\pi }}_{k}|n_{k}\right)\right],$$

where $N=(n_{1},n_{2},\ldots ,n_{K})$ is the observed data, $\hat{\gamma }=\left({\hat{\gamma }}_{1},{\hat{\gamma }}_{2},\ldots ,{\hat{\gamma }}_{k}\right)$ and $\hat{\pi }=\left({\hat{\pi }}_{1},{\hat{\pi }}_{2},\ldots ,{\hat{\pi }}_{k}\right)$ are the unconstrained MLEs, $\tilde{\gamma }$ and $\tilde{\pi }=\left({\tilde{\pi }}_{1},{\tilde{\pi }}_{2},\ldots ,{\tilde{\pi }}_{k}\right)$ are the constrained MLEs.

### 3.2. Score Statistic $T_{SC}$

Honda and Ohyama [19] proposed the score statistic. Denote

$$U={\left(\frac{\partial l_{1}}{\partial \gamma_{1}},\frac{\partial l_{2}}{\partial \gamma_{2}},\ldots ,\frac{\partial l_{K}}{\partial \gamma_{K}},0,0,\ldots ,0\right)}_{{}_{1\times 2K}}.$$

Under $H_{0}$, the score test statistic can be represented as

$$T_{SC}=UI_{2}^{-1}U^{T}{|}_{\gamma_{1}=\gamma_{2}=\cdots =\gamma_{K}=\tilde{\gamma },\pi =\tilde{\pi }},$$

where $\tilde{\gamma }$ and $\tilde{\pi }=({\tilde{\pi }}_{1},{\tilde{\pi }}_{2},\ldots ,$${\tilde{\pi }}_{K}{)}^{T}$ are the constrained MLEs. The 2K×2K Fisher information matrix $I_{2}$ is given in Appendix A.2. Through calculation, its simplified form is

$$T_{SC}=\sum_{k=1}^{K}\frac{r_{k}^{2}d_{k}}{n_{k}\left(b_{k}d_{k}-c_{k}^{2}\right)}{|}_{\gamma_{k}=\tilde{\gamma },\pi_{k}={\tilde{\pi }}_{k}},$$

where

$$\begin{array}{c} \begin{array}{cc} r_{k} & =\frac{n_{1k}}{P_{1k}\left(\gamma_{k},\pi_{k}\right)}-\frac{2n_{2k}}{P_{2k}\left(\gamma_{k},\pi_{k}\right)}+\frac{n_{3k}}{P_{3k}\left(\gamma_{k},\pi_{k}\right)},\\ b_{k} & =\frac{1}{P_{1k}\left(\gamma_{k},\pi_{k}\right)}+\frac{4}{P_{2k}\left(\gamma_{k},\pi_{k}\right)}+\frac{1}{P_{3k}\left(\gamma_{k},\pi_{k}\right)},\\ c_{k} & =\frac{1}{P_{1k}\left(\gamma_{k},\pi_{k}\right)}-\frac{1}{P_{3k}\left(\gamma_{k},\pi_{k}\right)}+\left(1-\gamma_{k}\right)\left(1-2\pi_{k}\right)b_{k},\\ d_{k} & =\frac{1}{P_{1k}\left(\gamma_{k},\pi_{k}\right)}+\frac{1}{P_{3k}\left(\gamma_{k},\pi_{k}\right)}+\left(1-\gamma_{k}\right)\left(1-2\pi_{k}\right)\left(\frac{1}{P_{1k}\left(\gamma_{k},\pi_{k}\right)}-\frac{1}{P_{3k}\left(\gamma_{k},\pi_{k}\right)}+c_{k}\right) \end{array} \end{array}$$

for k=1,2,…,K.

### 3.3. Wald-Type Statistic $T_{W}$

Denote $\beta ={\left(\gamma_{1},\gamma_{2},\ldots ,\gamma_{K},\pi_{1},\pi_{2},\ldots ,\pi_{K}\right)}_{{}_{1\times 2K}}$, and

$$C={\left[\begin{array}{cccccccccc} 1 & -1 & 0 & \cdots & \cdots & 0 & 0 & \cdots & \cdots & 0\\ 0 & 1 & -1 & \cdots & \cdots & 0 & 0 & \cdots & \cdots & 0\\ \vdots & \vdots & \vdots & \vdots & \vdots & \vdots & \vdots & \vdots & \vdots & \vdots \\ 0 & \cdots & \cdots & 0 & 1 & -1 & 0 & \cdots & \cdots & 0 \end{array}\right]}_{(K-1)\times 2K}.$$

The null hypothesis $H_{0}$ is equivalent to $C\beta^{T}=0$, where 0 is a zero vector. Thus, we define the Wald-type statistic as

$$T_{W}=\left(\beta C^{T}\right){\left(CI_{3}^{-1}C^{T}\right)}^{-1}\left(C\beta^{T}\right){|}_{\gamma_{k}={\hat{\gamma }}_{k},\pi_{k}={\hat{\pi }}_{k}},$$

where ${\hat{\gamma }}_{k}$ and ${\hat{\pi }}_{k}$ are the unconstrained MLEs. The Fisher information matrix $I_{3}$ is the same as that of the score test. We obtain the simplified form of $T_{W}$ as

$$T_{W}=\sum_{i=1}^{K-1}\sum_{j=1}^{K-1}\left({\hat{\gamma }}_{i}-{\hat{\gamma }}_{i+1}\right)\left({\hat{\gamma }}_{j}-{\hat{\gamma }}_{j+1}\right){\left(CI_{3}^{-1}C^{T}\right)}_{i,j}^{-1}.$$

Appendix A.3 provides the detailed process.

Under $H_{0}$, these three statistics $T_{L},T_{SC}$, and $T_{W}$ are asymptotically distributed as a chi-square distribution with K−1 degrees of freedom [34]. Given a significance level α, $H_{0}$ would be rejected if $T_{\theta }\geq \chi_{\left(K-1),(1-\alpha \right)}^{2},$ θ=L,SC,W, where $\chi_{\left(K-1),(1-\alpha \right)}^{2}$ is the 100(1−α) percentile of the chi-square distribution with K−1 degrees of freedom. For a special observed data $N^{*}=(n_{1},n_{2},\ldots ,$$n_{K})$, the *p*-values of these statistics are defined as

$$p_{\theta }^{A}\left(N^{*}\right)=P\left(\chi_{\left(K-1),(1-\alpha \right)}^{2}>T_{\theta }\left(N^{*}\right)\right),\;\theta =L,SC,W,$$

where $T_{\theta }\left(N^{*}\right)$ is the value of the statistic for the observed data $N^{*}$. For convenience, $p_{L}^{A},p_{SC}^{A}$, and $p_{W}^{A}$ are called asymptotic (A) approaches. Generally, asymptotic tests work well for large sample scenarios. However, they are conservative or liberal in the case of small sample sizes. Thus, we propose several exact methods based on the above statistics.

## 4. Exact Methods

Researchers often use the *p*-value to summarise the evidence against a null hypothesis. Thus, the key to the exact method is the calculation of the exact *p*-value. We uniformly denote the aforementioned test statistics $T_{L},T_{SC}$, and $T_{W}$ as $T_{\theta }$(θ=L,SC,W). Instead of relying on the chi-square distribution, the exact test can use the true sampling distribution of $T_{\theta }$ and compute an exact *p*-value. The calculation process is as follows. First, we need to generate all possible tables. For a given observed data $N^{*}$, the column margins $n_{1},n_{2},\ldots ,n_{K}$ are fixed. We enumerate all possible tables by varying the cell values. The detailed process is described as follows.

(i) Produce all possible values of each stratum, which is formed by all combinations $\left(n_{1k},n_{2k},n_{3k}\right)$ such that $n_{1k}+n_{2k}+n_{3k}=n_{k}\left(k=1,\ldots ,K\right)$, and $n_{k}$ is fixed. We take K=2 and $n_{1}=n_{2}=2$ as an example. There are six combinations in the *k*th stratum, including (0,0,2),(0,1,1),(0,2,0),(1,0,1),(1,1,0),(2,0,0).

(ii) Enumerate all possible tables determined by the combination of all strata. For K=2 and $n_{1}=n_{2}=2$, we can obtain 36 possible tables in Table 6.

Note that each column corresponds to a categorical table with *K* strata.

Through steps (i)–(ii), we can enumerate all possible tables for any observed data $N^{*}=\left(n_{1},n_{2},\ldots ,n_{K}\right)$. Then we identify the tail area from this reference set. The tail area includes all the tables whose statistic values equal or exceed the statistics of the observed data $N^{*}$. Finally, the exact *p*-value is calculated by summing the probabilities of all the tables in the tail area. The calculation of the exact *p*-value needs to eliminate the unknown parameters shown in the previous section. The following exact methods use different ways for the elimination of the unknown parameters.

### 4.1. E Approach

The E approach eliminates the unknown parameters by replacing them with the constrained MLEs. We first generate all possible tables. Define the tail area $\Omega_{E}\left(N^{*}\right)=\left\{N:T_{\theta }\left(N\right)\geq T_{\theta }\left(N^{*}\right)\right\}$ based on the test statistic $T_{\theta }$. The exact *p*-value of the observed data $N^{*}$ is expressed by

$$p_{\theta }^{E}\left(N^{*}\right)=P\left(T_{\theta }\left(N\right)\geq T_{\theta }\left(N^{*}\right)|{\tilde{\gamma }}^{*},{\tilde{\pi }}^{*}\right)=\sum_{N\in \Omega_{E}\left(N^{*}\right)}L\left({\tilde{\gamma }}^{*},{\tilde{\pi }}^{*}|N\right),\;\theta =L,SC,W,$$

where ${\tilde{\gamma }}^{*}$ and ${\tilde{\pi }}^{*}=\left({\tilde{\pi }}_{1}^{*},{\tilde{\pi }}_{2}^{*},\ldots ,{\tilde{\pi }}_{K}^{*}\right)$ are the constrained MLEs of γ and $\pi =(\pi_{1},\pi_{2},\ldots ,\pi_{K})$. Meanwhile, the probability of a table in the tail area is $L\left({\tilde{\gamma }}^{*},{\tilde{\pi }}^{*}|N\right)=\exp\left\{l_{0}\left({\tilde{\gamma }}^{*},{\tilde{\pi }}^{*}|N\right)\right\}$, which is the likelihood function under the null hypothesis. For convenience, $p_{L}^{E},p_{SC}^{E}$, and $p_{W}^{E}$ are collectively called the E approach.

### 4.2. M Approach

In Basu [30], the size of a test is always understood as the maximum probability of the type I error rate. Thus, the elimination of the unknown parameters for the M approach is to find the values of parameters over the whole range of γ and π, which can maximize the sum of probabilities of all the tables in the tail area. This maximum is the *p*-value of the M approach. Denote $\Theta =\{\pi :\pi_{k}\in \left[0,1\right],k=1,2,\ldots ,K\}$ and

$$\Lambda =\left\{\gamma :\frac{2-(1-|1-2\pi_{k}|)(3+|1-2\pi_{k}\left|\right)}{2-(1-|1-2\pi_{k}|)(1+|1-2\pi_{k}\left|\right)}\leq \gamma_{k}\leq 1\right\},$$

where $\pi =(\pi_{1},\pi_{2},\ldots ,\pi_{K})$ and $\gamma =(\gamma_{1},\gamma_{2},\ldots ,\gamma_{K})$. Similar to the E approach, the tail area can be calculated by $\Omega_{M}\left(N^{*}\right)=\left\{N:T_{\theta }\left(N\right)\geq T_{\theta }\left(N^{*}\right)\right\}$. Under these conditions, the exact *p*-value of the M approach can be defined as

$$p_{\theta }^{M}\left(N^{*}\right)=\underset{\gamma \in \Lambda ,\pi \in \Theta }{\sup}\left\{\sum_{N\in \Omega_{M}\left(N^{*}\right)}L\left(\gamma ,\pi |N\right)\right\},\;\;\theta =L,SC,W,$$

where $L\left(\gamma ,\pi |N\right)=\exp\left\{l(\gamma ,\pi |N)\right\}$ is the likelihood function under $H_{a}$. M approaches based on the three statistics are denoted as $p_{L}^{M},p_{SC}^{M}$, and $p_{W}^{M}$.

### 4.3. E+M Approach

The E approach is not always effective because of unsatisfactory type I error rates. Lloyd [31] used an additional maximization step to improve it, which is called the E+M approach. First, the *p*-value of the E approach is used as a test statistic to define the tail area. Then, we maximize the sum of probabilities of all the tables in the tail area as the exact *p*-value. Based on the above procedures, the tail area of the E+M approach is defined as $\Omega_{E+M}\left(N^{*}\right)=\left\{N:p_{\theta }^{E}\left(N\right)\leq p_{\theta }^{E}\left(N^{*}\right)\right\}$. The exact *p*-value of the E+M approach is expressed as

$$p_{\theta }^{EM}\left(N^{*}\right)=\underset{\gamma \in \Lambda ,\pi \in \Theta }{\sup}\left\{\sum_{N\in \Omega_{E+M}\left(N^{*}\right)}L\left(\gamma ,\pi |N\right)\right\},\;\;\theta =L,SC,W,$$

where L(γ,π|N) is the same as the likelihood function in the M approach. The E+M approach includes $p_{L}^{EM},p_{SC}^{EM}$, and $p_{W}^{EM}$.

## 5. Numerical Simulation

This section investigates the performance of asymptotic and exact methods in terms of type I error rates and powers. Given a significance level of 0.05, the type I error rate is the probability of rejecting $H_{0}$ when $H_{0}$ is true. According to Tang et al. [35], a test is considered liberal when the type I error rate is larger than 0.06, and conservative if it is less than 0.04; otherwise, it is robust. In several tables of this paper, we put the robust region of type I error rate (0.04–0.06) in bold to illustrate the performance of statistics. The power is defined by

$$power=1-\beta =1-P(H_{0}\;is\;accepted|H_{0}\;is\;false).$$

A test is optimal if it is robust and has more significant power.

### 5.1. Simulations of Asymptotic Methods

In the simulation, we first compare the performance of test statistics $T_{L},T_{SC}$, and $T_{W}$ in terms of empirical type I error rates under different parameter settings. Under $H_{0}:\gamma_{1}=\gamma_{2}=0.1$, we take K=2, $n_{1}=n_{2}=50$, and $\pi_{1}=\pi_{2}=0.3$ as an example to describe the detailed calculation process of empirical type I error rates.

(i) Bring the given values of $\gamma_{k}$, $n_{k}$, and $\pi_{k}\left(k=1,2\right)$ into (3). Let *F* be the cumulative distribution for the three types of ratings (l=1,2,3) of the two strata. Through calculation, we have
  $$\begin{array}{c} \left(\begin{array}{cc} P_{11} & P_{12}\\ P_{21} & P_{22}\\ P_{31} & P_{32} \end{array}\right)=\left(\begin{array}{cc} 0.0390 & 0.0390\\ 0.5220 & 0.5220\\ 0.4390 & 0.4390 \end{array}\right),F=\left(F_{lk}\right)=\left(\begin{array}{cc} 0.0390 & 0.0390\\ 0.5610 & 0.5610\\ 1.0000 & 1.0000 \end{array}\right). \end{array}$$
(ii) We produce an $n_{1}\times K$ pseudorandom matrix drawn from the standard uniform distribution on the open interval (0,1), denoted by $r=\left(r_{ik}\right)\left(i=1,2,\ldots ,n_{1},k=1,2,\ldots ,K\right)$. Define
  $$\begin{array}{c} n_{1k}=\sum_{i=1}^{n_{1}}I_{\left\{r_{ik}<F_{1k}\right\}},\;n_{3k}=\sum_{i=1}^{n_{1}}I_{\left\{r_{ik}>F_{2k}\right\}},\;k=1,2,\ldots ,K. \end{array}$$
  Then, $n_{2k}=n_{1}-n_{1k}-n_{3k}$ for k=1,2,…,K. When K=2, we can obtain a sample (or table) with two strata as follows:
  $$\begin{array}{c} \left(\begin{array}{cc} n_{11} & n_{12}\\ n_{21} & n_{22}\\ n_{31} & n_{32} \end{array}\right)=\left(\begin{array}{cc} 2 & 2\\ 23 & 26\\ 25 & 22 \end{array}\right). \end{array}$$
  When 10,000 pseudorandom matrices are given, 10,000 samples are randomly produced under the null hypothesis $H_{0}:\gamma_{1}=\gamma_{2}=0.1$.
(iii) For each sample, we calculate the corresponding MLEs and construct three statistics $T_{L},T_{SC}$, and $T_{W}$. Given a significance level α, $H_{0}$ would be rejected if $T_{\theta }\geq \chi_{\left(K-1),(1-\alpha \right)}^{2},$ θ=L,SC,W, where $\chi_{\left(K-1),(1-\alpha \right)}^{2}$ is the 100(1−α) percentile of the chi-square distribution with K−1 degrees of freedom.
(iv) The empirical type I error rate is calculated by the proportion of rejecting $H_{0}$, which is the number of rejections/10,000.

Through steps (i)–(iv), we can calculate the empirical type I error rates of asymptotic test statistics $T_{L},T_{SC}$, and $T_{W}$ under different parameter settings. In practice, the AC${}_{1}$ coefficient is usually positive. Under $H_{0}:\gamma_{1}=\ldots =\gamma_{K}≜\gamma$, Table 7 shows the empirical type I error rates of asymptotic statistics for K=2 under the balanced and unbalanced π settings. The corresponding results of K=3,4 are shown in Table A1 and Table A2 of the Appendix A.4. The tables show that the type I error rates of all the statistics are closer to the significance level of 0.05 with the increasing sample size. When the sample sizes are relatively small, the type I error rates of the likelihood ratio and score statistics are smaller than 0.05. The Wald-type test has a few liberal type I error rates. These three test statistics have conservative type I error rates for small and moderate samples when γ is close to 1. As the number of strata increases, $T_{W}$ becomes more liberal. For the unbalanced π, some type I error rates under γ=0.1 are more significant than 0.06 under K=3 and K=4. Overall, $T_{L}$ should be recommended because of the more robust type I error rates among the three statistics.

**Figure 2:**
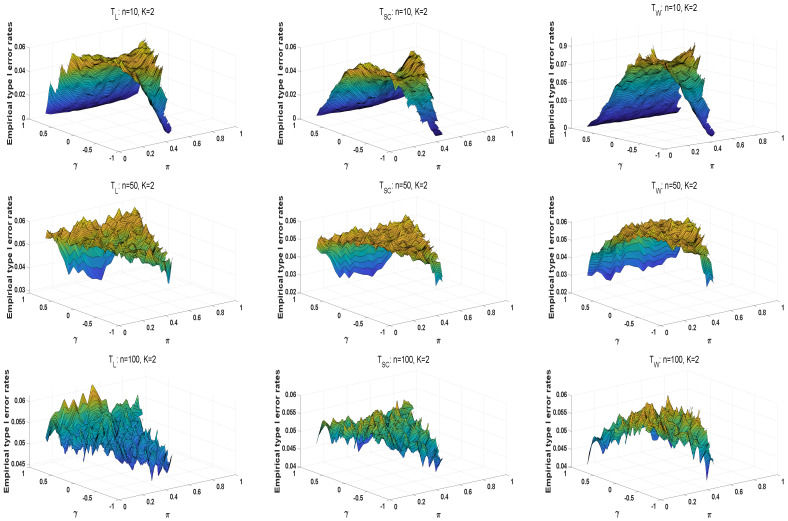
The surfaces of empirical type I error rates for asymptotic tests under K=2.

**Figure A1:**
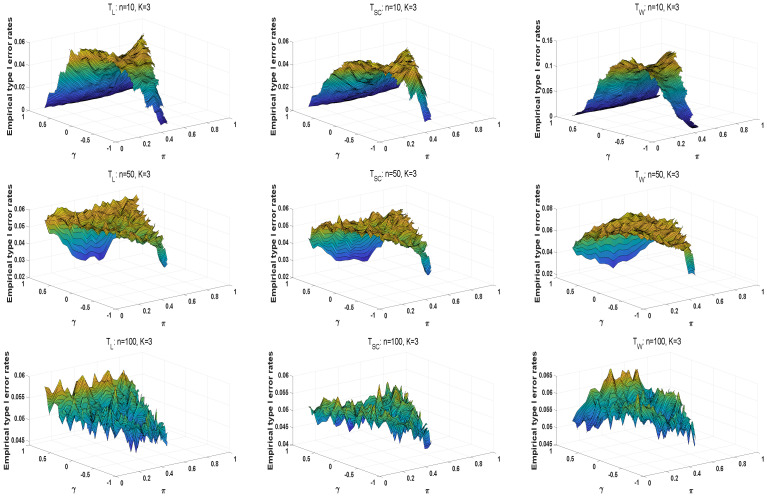
The surfaces of empirical type I error rates for asymptotic tests under K=3.

**Figure A2:**
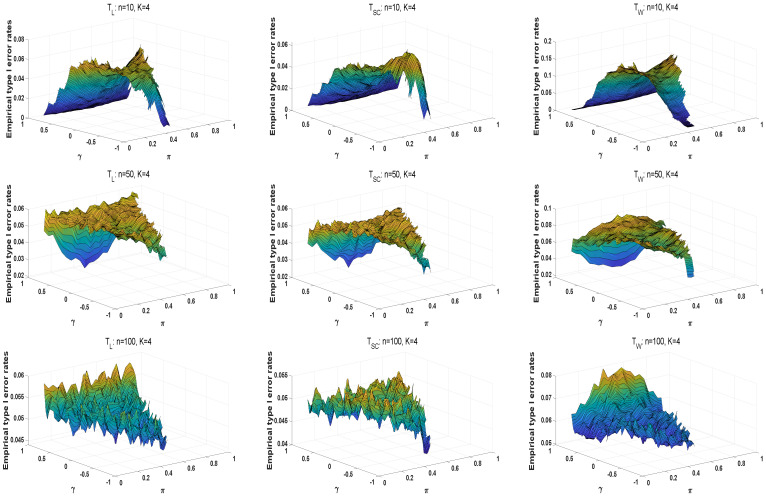
The surfaces of empirical type I error rates for asymptotic tests under K=4.

We use three-dimensional figures to investigate the asymptotic methods’ type I error rates. For convenience, the sample sizes are given as $n_{1}=n_{2}=\cdots =n_{K}≜n=10,50,100$. Parameters are selected from $\pi_{k}=\pi$ and $\gamma_{k}=\gamma \;\left(k=1,2,\ldots ,K,\;K=2,3,4\right)$. For each sample size, π increases from 0.1 to 0.9 by 0.04, and γ increases from −0.9 to 0.9 by 0.04. Figure 2 shows the distribution surfaces of type I error rates for all tests under K=2. Similarly, the cases of K=3,4 are displayed in Figure A1 and Figure A2 of the Appendix A.4. From these figures, we observe that the type I error rates of these statistics are smaller than 0.05 when the value of γ is close to −1 or 1. The type I error rates of Wald-type statistics tend to be larger for the same sample size with the increasing number of strata. Thus, it is more liberal than the other two statistics. The empirical type I error rates of the likelihood ratio and score statistics are closer to 0.05 as the sample size increases. For large sample scenarios, $T_{L}$, $T_{SC}$, and $T_{W}$ are usually robust, the type I error rates of $T_{L}$ are more concentrated at 0.05. Overall, the likelihood ratio statistic performs better under all configurations. However, when the sample sizes are small, most type I error rates of $T_{L}$ and $T_{SC}$ are smaller than 0.04, and those of $T_{W}$ are greater than 0.06.

**Figure 3:**
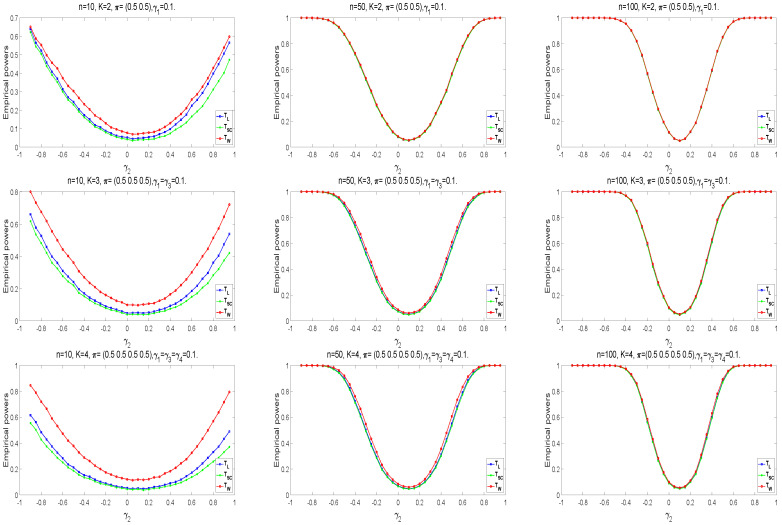
Empirical power curves of asymptotic tests for K=2,3,4.

Next, we analyze the powers of the proposed tests, which are similar to the calculation of empirical type I error rate when the samples are generated from the alternative hypothesis $H_{a}$. Take $n_{1}=n_{2}=\cdots =n_{K}≜n=10,50,100$. The following parameter settings are considered for each sample size: (i) K=2, π=(0.5,0.5), $\gamma_{1}=0.1$, $\gamma_{2}=-0.9:0.05:0.95$, (ii) $K=3,\pi =\left(0.5,0.5,0.5\right),\gamma_{1}=\gamma_{3}=0.1,\gamma_{2}=-0.9:0.05:0.95$, (iii) $K=4,\pi =\left(0.5,0.5,0.5,0.5\right),\gamma_{1}=\gamma_{3}=\gamma_{4}=0.1,\gamma_{2}=-0.9:0.05:0.95$. Here, a:b:c means increasing from *a* to *c* by *b*. Under the alternative hypothesis $H_{a}$, we randomly generate 10,000 samples for each design. The empirical power equals the proportion of rejecting $H_{0}$ in all samples. Figure 3 shows the empirical powers of the three asymptotic tests. The Wald-type test has higher empirical powers than the other two statistics, especially in small samples. The powers of test statistics become higher if there exists a more considerable difference between $\gamma_{2}$ and $\gamma_{1}\;\left(\gamma_{3}\right)$. Among these statistics, the values of powers are closer as the sample size becomes larger.

Above all, $T_{L}$ is recommended for the homogeneity test of stratified AC${}_{1}$ in large sample sizes because of the robust type I error rates and satisfactory powers.

### 5.2. Exact Methods Results

**Figure 4:**
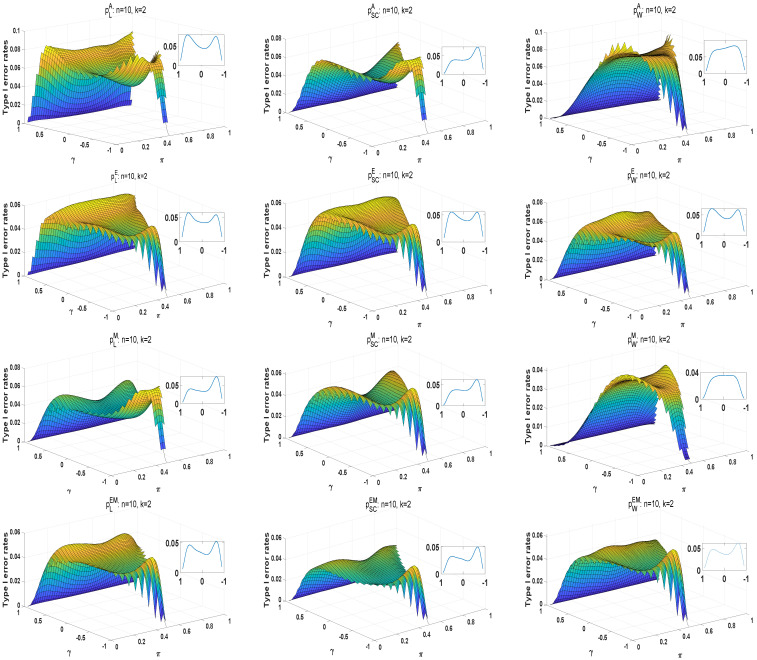
The surfaces and curves of type I error rates for exact methods under K=2.

**Figure A3:**
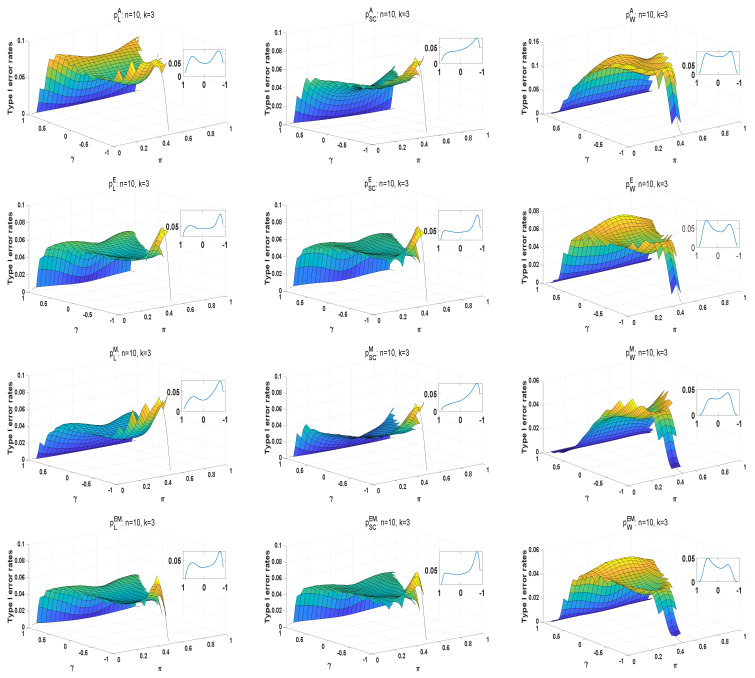
The surfaces and curves of type I error rates for exact methods under K=3.

Considering the unsatisfactory performance of asymptotic methods in small sample sizes, we introduce the exact E, M, and E+M methods to improve effectiveness. The type I error rates and powers of these three methods are compared with asymptotic approaches to investigate the advantages of exact methods. The algorithm for exact *p*-value is usually computationally intensive, time consuming, and sometimes exceeds the memory limits of the computer. Thus, running time is an important determinant for the appropriate numbers of strata and sample size in the numerical study of exact methods. For simplicity, we only focus on $n_{1}=\cdots =n_{k}≜n$, $\pi_{k}=0.5$, and $\gamma_{k}=0.1\;\left(k=1,2,\ldots ,K,\right)$. The average of 100 running times is used as the running time of the exact *p*-value. We study the running times in the case of the following parameter settings: (i) K=2, n=2,…,11, and (ii) n=3, K=2,3,4,5. The running times of different methods are shown in Table 8. From the results, it is obvious that running time will increase exponentially for the growth of the number of strata *K* and the marginal numbers *n*. It is challenging for a computer’s megabytes of storage and clock speed. Thus, the cases n=10 and K=2,3 are considered in our work. Take $\pi_{k}=\pi$, $\gamma_{k}=\gamma \;\left(k=1,2,\ldots ,K,\right)$, where π=0:0.02:1, and γ=−1:0.02:1. Figure 4 shows the surfaces of type I error rates for K=2. In the Appendix A.4, we provide the case of K=3 in Figure A3. The small diagrams in the upper right corner reflect the curves of the type I error rates under π=0.5 and γ=−1:0.02:1. For the large diagrams, $p_{L}^{A}$ and $p_{W}^{A}$ have liberal type I error rates, while the type I error rates of $p_{SC}^{A}$ are smaller than 0.05. The M and E+M approaches produce conservative type I error rates. The E approaches under the likelihood ratio and score statistics are better than that under the Wald-type test when K=2. For K=3, the surfaces of E approaches under three statistics are closer to the significance level in the case of positive γ. From small diagrams, the curves of type I error rates have bimodal shapes. To reveal the reason, we consider the case of $K=2,\gamma_{k}=\gamma$, and $\pi_{k}=0.5$. As a part of tail probability,

$$LL=\exp\left(\sum_{k=1}^{K}\left(l_{k}\left(\gamma_{k},\pi_{k}\right)|n_{k}\right)\right)$$

has the same value for each γ under the fixed sum of $n_{2k}$. Table 9 reflects the changes of LL as the increases of γ and ($n_{21}+n_{22}$). The table shows that the increase of γ can affect the peak change with the given sum of $n_{2k}$. The peak also occurs when the sum of $n_{2k}$ increases for a given γ. Meanwhile, each method’s tail area determines the bimodal shape’s location and values. Next, we compare the type I error rates of exact and asymptotic methods under several parameters. Let K=2 and $n_{1}=n_{2}=10,25$. From Table 10, the type I error rates of $p_{L}^{E}$ and $p_{SC}^{E}$ are closer to 0.05 than those of $p_{W}^{E}$. The E approach works better as the sample size increases in the range of small sample sizes. The type I error rates of E approaches are close to 0.05 when $n_{1}=n_{2}=25$. It reveals that the E approach is more effective than asymptotic methods for high γ.

**Figure 5:**
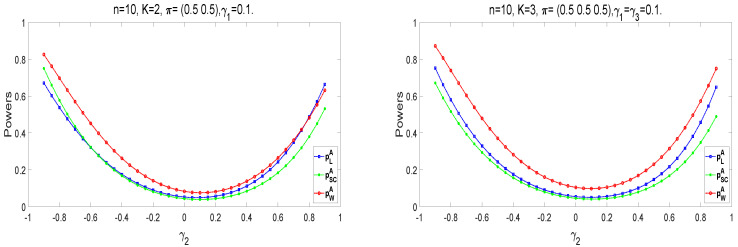

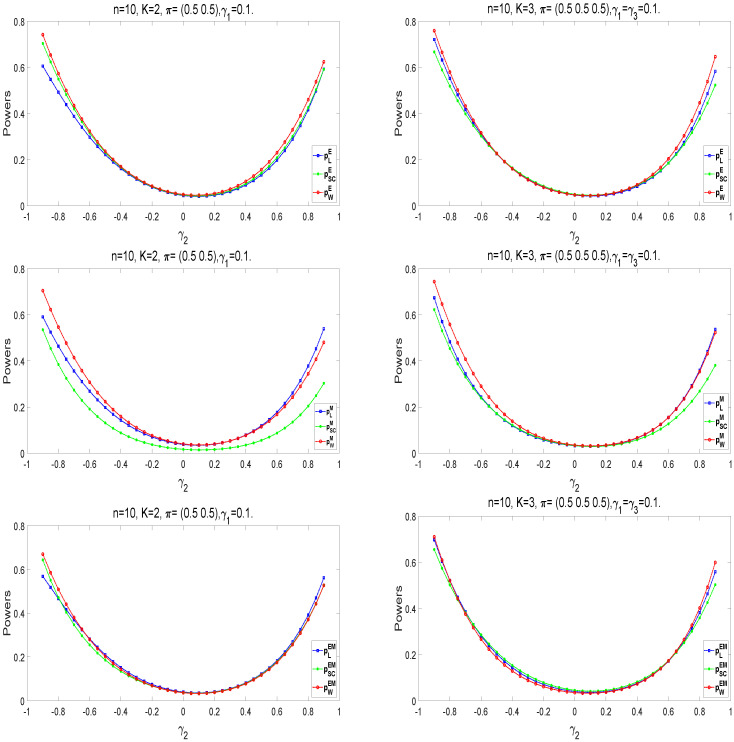
Power curves of exact methods for K=2,3.

According to the relationship between $\gamma_{k}$ and $\pi_{k}$, the parameter settings are considered under $H_{a}$ as follows: (i) $n_{k}=n=10,K=2,\pi =\left(0.5,0.5\right),\gamma_{1}=0.1,\gamma_{2}=-0.9:0.05:0.9$; (ii) $n_{k}=n=10,K=3,\pi =\left(0.5,0.5,0.5\right),\gamma_{1}=\gamma_{3}=0.1,\gamma_{2}=-0.9:0.05:0.9,\;\left(k=1,2,\ldots ,K\right)$. Figure 5 provides the power curves of the exact methods. For the A approach, $p_{W}^{A}$ has higher powers under different parameter settings. The power becomes larger as the absolute value of $\gamma_{2}-\gamma_{1}\;\left(\gamma_{3}\right)$ becomes larger. On the contrary, the powers of each method tend to be 0.05 as $\gamma_{2}$ becomes closer to 0.1. The power curves of the E+M approaches are close to each other. Then, we compare the powers of asymptotic and exact methods under different parameter settings. For K=2 and $n_{1}=n_{2}=\cdots =n_{K}=10$, the values of $\gamma_{k}$ are taken as (i) $\gamma_{1}=0.1,\gamma_{2}=0.5:0.1:0.9$; (ii) $\gamma_{1}=0.3,\gamma_{2}=0.6:0.1:0.9$; and (iii) $\gamma_{1}=0.5,\gamma_{2}=0.8,0.9$. When K=3, let $\gamma_{1}=\gamma_{3}$ and other settings be the same. In Table 11, we provide the power comparisons of K=2 under the balanced π conditions. Table A3, Table A4 and Table A5 show the comparisons of these methods under other settings. The powers of exact methods are generally smaller than those of asymptotic methods. However, $p_{SC}^{E}$ has higher powers than $p_{SC}^{A}$. For exact methods, the E approach has higher power than the other two methods, in which the E approach under the Wald-type statistic has higher power.

In summary, the exact E method can effectively improve the effectiveness of the homogeneity test of stratified AC${}_{1}$ under small sample sizes and high γ. The E approaches under the likelihood ratio and score statistics perform better than that under the Wald-type statistic.

## 6. Applications

We review the two real examples in the introduction. Table 3 shows the data structure among 83 twins. For the large sample size, the hypotheses of the homogeneity test for AC${}_{1}$ across two strata are

$$H_{0}:\gamma_{1}=\gamma_{2}≜\gamma \;\;\;\mathrm{vs}\;\;\;H_{a}:\gamma_{i}\;\mathrm{is}\;\mathrm{not}\;\mathrm{all}\;\mathrm{the}\;\mathrm{same},\;\;\;i=1,2.$$

By computation, the unconstrained MLEs of γ and π are $\hat{\gamma }=\left(0.4615,-0.0312\right)$ and $\hat{\pi }$=(0.5000,0.5161). The constrained MLEs of γ and π are $\tilde{\gamma }=0.2788$ and $\tilde{\pi }$=(0.5000,0.5351). Table 12 provides the results of statistics and *p*-values. Given a significance level α=0.05, the values of test statistics $T_{L},T_{SC},T_{W}$ are all larger than $\chi_{1,0.95}^{2}=3.8415$. All the *p*-values of these three tests are smaller than 0.05. Thus, the null hypothesis $H_{0}$ is rejected at the significance level 0.05. There is a significant difference between two AC${}_{1}$ coefficients, and we cannot merge the data of two strata to compute a common coefficient. Then, we need to investigate how zygosity affects the consistency of the alcohol-drinking status of male twins.

Table 4 shows the small data structure for the clinical COVID-19 trial. The A, E, M, and E+M methods are applied to test $H_{0}:\gamma_{1}=\gamma_{2}≜\gamma \;\;\;\mathrm{vs}\;\;\;H_{a}:\gamma_{1}\neq \gamma_{2}.$ By calculation, the unconstrained MLEs of parameters γ and π are $\hat{\gamma }=\left(0.6656,0.2197\right)$ and $\hat{\pi }=\left(0.6176,0.6176\right)$. The constrained MLEs are $\tilde{\gamma }=0.4537$ and $\tilde{\pi }=\left(0.5882,0.6666\right)$. The values of statistics are $T_{L}\left(N^{*}\right)=2.0150,\;T_{SC}\left(N^{*}\right)=1.9674$, and $T_{W}\left(N^{*}\right)=2.0805$. Table 13 shows the corresponding *p*-values of asymptotic and exact methods. The running time is about 8 min because of two strata and $n_{1}=n_{2}=17$, referred to Table 8. There is no significant difference in stratified AC${}_{1}$ for any approaches. Thus, we can merge the data in these two strata and estimate the value of common AC${}_{1}$ by MLEs, which is 0.4537.

## 7. Concluding Remarks

This article defines the stratified AC${}_{1}$ coefficients as the object of study and constructs the likelihood function of the observed data. The primary purpose is to derive various statistics for testing the homogeneity of stratified AC${}_{1}$ in the case of two raters with a binary outcome. We constructed asymptotic and exact methods for large and small sample sizes. Two asymptotic test statistics and their explicit expressions are derived for large sample sizes, including the likelihood ratio statistic ($T_{L}$) and Wald-type statistic ($T_{W}$). Meanwhile, the score statistic ($T_{SC}$) proposed by Honda and Ohyama [19] is also reviewed. Asymptotic *p*-values $p_{\theta }^{A}$(θ=L,SC,W) under the statistics mentioned above are denoted as the A approach. Three exact methods (E, M, and E+M) are proposed based on $T_{L},T_{SC}$, and $T_{W}$ for small sample sizes.

We conduct numerical studies to compare the performance of the above methods by type I error rates and powers. For large samples, the type I error rates of the likelihood ratio statistic are closer to the predetermined significance level of 0.05. The powers of statistics are better as the sample size increases. Overall, the likelihood ratio statistic is optimal among these three statistics in large sample sizes. However, asymptotic tests may generate unsatisfactory type I error rates at small sample scenarios and high γ. For small sample sizes in K=2, $p_{L}^{A}$ and $p_{W}^{A}$ are liberal, and $p_{SC}^{A}$ has the conservative type I error rates under different parameter settings. The type I error rates of the E approach are closer to the significance level of 0.05. The M and E+M approaches have conservative type I error rates. Moreover, $p_{L}^{E}$ and $p_{SC}^{E}$ are more robust than $p_{W}^{E}$. When K=3, and γ is positive, the E approach has the robust type I error rates among three methods, in which $p_{L}^{E}$ and $p_{SC}^{E}$ perform better. The type I error rates of the exact E method are closer to 0.05 as the sample size increases. The E approach can improve the effectiveness of homogeneity tests at high γ. In the case of powers, the A approach has larger powers than these exact methods. Under the Wald-type statistic, each exact method has a higher power. Thus, the E approaches based on the likelihood ratio and score statistics have a robust performance for small sample sizes.

The proposed methods can be used not only in medical research but also in biometrics and psychological measurements. Meanwhile, exact methods can be applied to other data types, such as binary outcomes on multiple raters. This work focuses on constructing parametric statistics through unconstrained and constrained MLEs. However, there are still many problems that need to be solved. For example, how to construct optimal tests? How to improve exact methods effectively for a larger *K* or sample size $n_{k}$? How to simplify the heavy computations caused by the consideration of all the tables? More studies should be conducted on these problems and be extended in the future.

## Figures and Tables

**Table 1:** A 2×2 original table.

Rater		Rater A		Total
		+	−	
	+	$n_{11}\left(P_{11}\right)$	$n_{12}\left(P_{12}\right)$	$n_{1+}\left(P_{B}\right)$
Rater B	−	$n_{21}\left(P_{21}\right)$	$n_{22}\left(P_{22}\right)$	$n_{2+}\left(1-P_{B}\right)$
Total		$n_{+1}\left(P_{A}\right)$	$n_{+2}\left(1-P_{A}\right)$	*n*

**Table 2:** A 2×2 table under $P_{A}=P_{B}$.

Category	Ratings	Frequency	Probability
1	(+,+)	$n_{11}$	$P_{11}$
2	(+,−) or (−,+)	$n_{12}+n_{21}$	$2P_{12}$
3	(−,−)	$n_{22}$	$P_{22}$
Total		*n*	1

**Table 3:** Agreement of alcohol drinking status between male twins stratified by zygosity.

Alcohol Drinking	Zygosity	
	MZ	DZ
Both	19	8
One	14	16
Neither	19	7
Total	52	31

Note: Both: (twin 1, twin 2) = (drinker, drinker); One: (twin 1, twin 2) = (drinker, nondrinker) or (nondrinker, drinker); neither: (twin 1, twin 2) = (nondrinker, nondrinker).

**Table 4:** Agreement between ELISA and gold-standard methods stratified by antibody type.

Number of Agreement	Antibody Type	
	IgG	IgM
Both	9	7
One	3	7
Neither	5	3
Total	17	17

Note: Both: (ELISA, gold-standard) = (+,+); One: (ELISA, gold-standard) = (+,−) or (−,+); neither: (ELISA, gold-standard) = (−,−).

**Table 5:** Frequencies and probabilities of ratings in *K* strata.

Category	Ratings	Frequency of Subjects				Total
		Stratum 1	Stratum 2	⋯	Stratum *K*	
1	(+,+)	$n_{11}\;\left(P_{11}\left(\gamma_{1},\pi_{1}\right)\right)$	$n_{12}\;\left(P_{12}\left(\gamma_{2},\pi_{2}\right)\right)$	⋯	$n_{1K}\;\left(P_{1K}\left(\gamma_{K},\pi_{K}\right)\right)$	$S_{1}$
2	(+,−) or (−,+)	$n_{21}\;\left(P_{21}\left(\gamma_{1},\pi_{1}\right)\right)$	$n_{22}\;\left(P_{22}\left(\gamma_{2},\pi_{2}\right)\right)$	⋯	$n_{2K}\;\left(P_{2K}\left(\gamma_{K},\pi_{K}\right)\right)$	$S_{2}$
3	(−,−)	$n_{31}\;\left(P_{31}\left(\gamma_{1},\pi_{1}\right)\right)$	$n_{32}\;\left(P_{32}\left(\gamma_{2},\pi_{2}\right)\right)$	⋯	$n_{3K}\;\left(P_{3K}\left(\gamma_{K},\pi_{K}\right)\right)$	$S_{3}$
Total		$n_{1}$	$n_{2}$	⋯	$n_{K}$	*N*

**Table 6:** All the possible tables for K=2 and $n_{1}=n_{2}=2$.

$n_{1}$	$n_{11}$	0	0	0	0	0	0	0	0	0	0	0	0	0	0	0	0	0	0	1	1	1	1	1	1	1	1	1	1	1	1	2	2	2	2	2	2
	$n_{21}$	0	0	0	0	0	0	1	1	1	1	1	1	2	2	2	2	2	2	0	0	0	0	0	0	1	1	1	1	1	1	0	0	0	0	0	0
	$n_{31}$	2	2	2	2	2	2	1	1	1	1	1	1	0	0	0	0	0	0	1	1	1	1	1	1	0	0	0	0	0	0	0	0	0	0	0	0
$n_{2}$	$n_{12}$	0	0	0	1	1	2	0	0	0	1	1	2	0	0	0	1	1	2	0	0	0	1	1	2	0	0	0	1	1	2	0	0	0	1	1	2
	$n_{22}$	0	1	2	0	1	0	0	1	2	0	1	0	0	1	2	0	1	0	0	1	2	0	1	0	0	1	2	0	1	0	0	1	2	0	1	0
	$n_{32}$	2	1	0	1	0	0	2	1	0	1	0	0	2	1	0	1	0	0	2	1	0	1	0	0	2	1	0	1	0	0	2	1	0	1	0	0

**Table 7:** Empirical type I error rates of asymptotic statistics for K=2 (balanced sample sizes).

Balanced π Conditions						Unbalanced π Conditions						
$n_{1}=n_{2}$	γ	$\pi_{1}=\pi_{2}$	$T_{L}$	$T_{\mathrm{SC}}$	$T_{W}$	$n_{1}=n_{2}$	γ	$\pi_{1}$	$\pi_{2}$	$T_{L}$	$T_{\mathrm{SC}}$	$T_{W}$
10	0.1	0.3	0.0536	0.0456	0.0789	10	0.1	0.3	0.5	0.0526	0.0425	0.0787
	0.3		0.0562	0.0483	0.0753		0.3			0.0506	0.0403	0.0702
	0.5		0.0434	0.0363	0.0540		0.5			0.0445	0.0326	0.0541
	0.7		0.0233	0.0186	0.0228		0.7			0.0192	0.0129	0.0225
	0.9		0.0018	0.0016	0.0018		0.9			0.0014	0.0007	0.0013
	0.1	0.6	0.0522	0.0452	0.0764		0.1	0.6	0.4	0.0521	0.0431	0.0750
	0.3		0.0515	0.0397	0.0697		0.3			0.0490	0.0362	0.0645
	0.5		0.0384	0.0264	0.0461		0.5			0.0440	0.0271	0.0513
	0.7		0.0189	0.0110	0.0227		0.7			0.0215	0.0127	0.0250
	0.9		0.0005	0.0002	0.0006		0.9			0.0009	0.0005	0.0013
	0.1	0.5	0.0483	0.0386	0.0727		0.1	0.5	0.4	0.0504	0.0422	0.0770
	0.3		0.0513	0.0335	0.0653		0.3			0.0504	0.0342	0.0670
	0.5		0.0424	0.0238	0.0496		0.5			0.0453	0.0276	0.0530
	0.7		0.0161	0.0080	0.0194		0.7			0.0140	0.0071	0.0173
	0.9		0.0006	0.0003	0.0012		0.9			0.0011	0.0005	0.0013
50	0.1	0.3	0.0496	0.0480	0.0558	50	0.1	0.3	0.5	0.0539	0.0526	0.0601
	0.3		0.0525	0.0515	0.0554		0.3			0.0544	0.0528	0.0576
	0.5		0.0524	0.0512	0.0542		0.5			0.0547	0.0537	0.0565
	0.7		0.0531	0.0493	0.0500		0.7			0.0529	0.0489	0.0515
	0.9		0.0394	0.0309	0.0280		0.9			0.0330	0.0219	0.0221
	0.1	0.6	0.0511	0.0504	0.0567		0.1	0.6	0.4	0.0498	0.0495	0.0549
	0.3		0.0480	0.0470	0.0521		0.3			0.0539	0.0525	0.0572
	0.5		0.0512	0.0490	0.0533		0.5			0.0518	0.0503	0.0544
	0.7		0.0552	0.0515	0.0545		0.7			0.0550	0.0505	0.0539
	0.9		0.0366	0.0269	0.0310		0.9			0.0375	0.0297	0.0330
	0.1	0.5	0.0493	0.0482	0.0517		0.1	0.5	0.4	0.0500	0.0491	0.0539
	0.3		0.0472	0.0464	0.0508		0.3			0.0516	0.0500	0.0540
	0.5		0.0506	0.0490	0.0532		0.5			0.0506	0.0487	0.0531
	0.7		0.0516	0.0471	0.0519		0.7			0.0535	0.0485	0.0531
	0.9		0.0326	0.0256	0.0310		0.9			0.0345	0.0248	0.0291
100	0.1	0.3	0.0493	0.0484	0.0520	100	0.1	0.3	0.5	0.0514	0.0508	0.0545
	0.3		0.0494	0.0492	0.0506		0.3			0.0473	0.0466	0.0490
	0.5		0.0516	0.0512	0.0524		0.5			0.0507	0.0499	0.0520
	0.7		0.0489	0.0476	0.0478		0.7			0.0527	0.0504	0.0523
	0.9		0.0547	0.0463	0.0440		0.9			0.0564	0.0493	0.0503
	0.1	0.6	0.0521	0.0518	0.0541		0.1	0.6	0.4	0.0483	0.0482	0.0514
	0.3		0.0493	0.0486	0.0518		0.3			0.0499	0.0495	0.0514
	0.5		0.0522	0.0514	0.0528		0.5			0.0509	0.0498	0.0520
	0.7		0.0530	0.0505	0.0522		0.7			0.0517	0.0496	0.0514
	0.9		0.0576	0.0463	0.0476		0.9			0.0568	0.0455	0.0466
	0.1	0.5	0.0473	0.0472	0.0489		0.1	0.5	0.4	0.0512	0.0510	0.0536
	0.3		0.0463	0.0461	0.0475		0.3			0.0552	0.0544	0.0568
	0.5		0.0523	0.0513	0.0533		0.5			0.0513	0.0503	0.0520
	0.7		0.0510	0.0489	0.0508		0.7			0.0568	0.0539	0.0568
	0.9		0.0579	0.0407	0.0457		0.9			0.0583	0.0478	0.0494

Note: The type I error rates of robust region (0.04–0.06) are shown in bold.

**Table 8:** Running times (seconds) for γ=0.1 and π=0.5.

Value	*n*										*K*			
	2	3	4	5	6	7	8	9	10	11	2	3	4	5
$p_{L}^{E}$	0.0088	0.0278	0.0540	0.1416	0.3046	0.6356	1.1910	2.2842	3.9241	7.6825	0.0278	1.0149	33.7988	3743.0157
$p_{SC}^{E}$	0.0115	0.0324	0.0692	0.1778	0.3651	0.7224	1.2988	2.4110	4.3842	8.4029	0.0324	0.9989	37.1695	3814.3481
$p_{W}^{E}$	0.0125	0.0364	0.0837	0.2092	0.4009	0.8020	1.4019	2.5217	4.5078	8.4676	0.0364	0.8794	39.2842	3797.6383
$p_{L}^{M}$	0.2946	0.4833	0.8561	1.7686	4.1932	8.1121	11.5237	16.2005	22.9323	34.0228	0.4833	5.9487	86.4241	1584.3279
$p_{SC}^{M}$	0.2867	0.5865	0.9794	2.1689	4.5669	7.8500	11.4646	17.1822	23.6388	42.0095	0.5865	5.3177	58.3290	1237.8588
$p_{W}^{M}$	0.2608	0.5477	0.9320	2.2620	4.1170	6.6121	10.8523	14.0822	24.4280	37.1586	0.5477	6.4484	109.0489	990.4258
$p_{L}^{EM}$	0.3457	0.7130	1.1282	2.3653	4.3037	8.1787	13.6343	18.7686	30.1321	49.2284	0.7130	7.9202	159.2139	5696.1438
$p_{SC}^{EM}$	0.3446	0.6777	1.0412	2.2872	4.3691	8.0231	12.8295	20.0811	30.1819	45.1356	0.6777	6.2942	131.3634	4930.5572
$p_{W}^{EM}$	0.3745	0.7553	1.1046	2.6653	4.6391	8.3511	12.5336	17.4634	29.6501	47.6976	0.7553	6.9710	186.0982	6903.8451

**Table 9:** The values of LL in different settings for K=2.

$n_{21}+n_{22}$	γ																		
	−0.9	−0.8	−0.7	−0.6	−0.5	−0.4	−0.3	−0.2	−0.1	0	0.1	0.2	0.3	0.4	0.5	0.6	0.7	0.8	0.9
2	1.31 × $10^{-29}$	3.09 × $10^{-24}$	4.07 × $10^{-21}$	6.40 × $10^{-19}$	3.12 × $10^{-17}$	7.24 × $10^{-16}$	1.00 × $10^{-14}$	9.44 × $10^{-14}$	6.61 × $10^{-13}$	3.64 × $10^{-12}$	1.64 × $10^{-11}$	6.20 × $10^{-11}$	2.00 × $10^{-10}$	5.59 × $10^{-10}$	1.34 × $10^{-09}$	2.75 × $10^{-09}$	4.60 × $10^{-09}$	5.73 × $10^{-09}$	3.79 × $10^{-09}$
3	4.99 × $10^{-28}$	5.56 × $10^{-23}$	4.62 × $10^{-20}$	5.12 × $10^{-18}$	1.87 × $10^{-16}$	3.38 × $10^{-15}$	3.72 × $10^{-14}$	2.83 × $10^{-13}$	1.62 × $10^{-12}$	7.28 × $10^{-12}$	2.68 × $10^{-11}$	8.27 × $10^{-11}$	2.16 × $10^{-10}$	4.79 × $10^{-10}$	8.96 × $10^{-10}$	1.37 × $10^{-09}$	1.63 × $10^{-09}$	1.27 × $10^{-09}$	3.99 × $10^{-10}$
4	1.90 × $10^{-26}$	1.00 × $10^{-21}$	5.23 × $10^{-19}$	4.10 × $10^{-17}$	1.12 × $10^{-15}$	1.58 × $10^{-14}$	1.38 × $10^{-13}$	8.49 × $10^{-13}$	3.95 × $10^{-12}$	1.46 × $10^{-11}$	4.39 × $10^{-11}$	1.10 × $10^{-10}$	2.32 × $10^{-10}$	4.11 × $10^{-10}$	5.97 × $10^{-10}$	6.87 × $10^{-10}$	5.74 × $10^{-10}$	2.83 × $10^{-10}$	4.20 × $10^{-11}$
5	7.21 × $10^{-25}$	1.80 × $10^{-20}$	5.93 × $10^{-18}$	3.28 × $10^{-16}$	6.74 × $10^{-15}$	7.36 × $10^{-14}$	5.13 × $10^{-13}$	2.55 × $10^{-12}$	9.65 × $10^{-12}$	2.91 × $10^{-11}$	7.18 × $10^{-11}$	1.47 × $10^{-10}$	2.50 × $10^{-10}$	3.52 × $10^{-10}$	3.98 × $10^{-10}$	3.44 × $10^{-10}$	2.02 × $10^{-10}$	6.28 × $10^{-11}$	4.42 × $10^{-12}$
6	2.74 × $10^{-23}$	3.24 × $10^{-19}$	6.72 × $10^{-17}$	2.62 × $10^{-15}$	4.05 × $10^{-14}$	3.43 × $10^{-13}$	1.91 × $10^{-12}$	7.64 × $10^{-12}$	2.36 × $10^{-11}$	5.82 × $10^{-11}$	1.17 × $10^{-10}$	1.96 × $10^{-10}$	2.70 × $10^{-10}$	3.02 × $10^{-10}$	2.66 × $10^{-10}$	1.72 × $10^{-10}$	7.14 × $10^{-11}$	1.40 × $10^{-11}$	4.65 × $10^{-13}$
7	1.04 × $10^{-21}$	5.84 × $10^{-18}$	7.62 × $10^{-16}$	2.10 × $10^{-14}$	2.43 × $10^{-13}$	1.60 × $10^{-12}$	7.08 × $10^{-12}$	2.29 × $10^{-11}$	5.77 × $10^{-11}$	1.16 × $10^{-10}$	1.92 × $10^{-10}$	2.61 × $10^{-10}$	2.90 × $10^{-10}$	2.59 × $10^{-10}$	1.77 × $10^{-10}$	8.59 × $10^{-11}$	2.52 × $10^{-11}$	3.10 × $10^{-12}$	4.90 × $10^{-14}$
8	3.95 × $10^{-20}$	1.05 × $10^{-16}$	8.63 × $10^{-15}$	1.68 × $10^{-13}$	1.46 × $10^{-12}$	7.48 × $10^{-12}$	2.63 × $10^{-11}$	6.88 × $10^{-11}$	1.41 × $10^{-10}$	2.33 × $10^{-10}$	3.15 × $10^{-10}$	3.48 × $10^{-10}$	3.13 × $10^{-10}$	2.22 × $10^{-10}$	1.18 × $10^{-10}$	4.30 × $10^{-11}$	8.90 × $10^{-12}$	6.90 × $10^{-13}$	5.15 × $10^{-15}$
9	1.50 × $10^{-18}$	1.89 × $10^{-15}$	9.78 × $10^{-14}$	1.34 × $10^{-12}$	8.74 × $10^{-12}$	3.49 × $10^{-11}$	9.76 × $10^{-11}$	2.06 × $10^{-10}$	3.45 × $10^{-10}$	4.66 × $10^{-10}$	5.15 × $10^{-10}$	4.64 × $10^{-10}$	3.37 × $10^{-10}$	1.90 × $10^{-10}$	7.87 × $10^{-11}$	2.15 × $10^{-11}$	3.14 × $10^{-12}$	1.53 × $10^{-13}$	5.42 × $10^{-16}$
10	5.71 × $10^{-17}$	3.41 × $10^{-14}$	1.11 × $10^{-12}$	1.07 × $10^{-11}$	5.24 × $10^{-11}$	1.63 × $10^{-10}$	3.63 × $10^{-10}$	6.19 × $10^{-10}$	8.42 × $10^{-10}$	9.31 × $10^{-10}$	8.42 × $10^{-10}$	6.19 × $10^{-10}$	3.63 × $10^{-10}$	1.63 × $10^{-10}$	5.24 × $10^{-11}$	1.07 × $10^{-11}$	1.11 × $10^{-12}$	3.41 × $10^{-14}$	5.71 × $10^{-17}$
11	2.17 × $10^{-15}$	6.13 × $10^{-13}$	1.26 × $10^{-11}$	8.59 × $10^{-11}$	3.15 × $10^{-10}$	7.60 × $10^{-10}$	1.35 × $10^{-09}$	1.86 × $10^{-09}$	2.06 × $10^{-09}$	1.86 × $10^{-09}$	1.38 × $10^{-09}$	8.26 × $10^{-10}$	3.91 × $10^{-10}$	1.40 × $10^{-10}$	3.50 × $10^{-11}$	5.37 × $10^{-12}$	3.91 × $10^{-13}$	7.57 × $10^{-15}$	6.01 × $10^{-18}$
12	8.25 × $10^{-14}$	1.10 × $10^{-11}$	1.42 × $10^{-10}$	6.87 × $10^{-10}$	1.89 × $10^{-09}$	3.55 × $10^{-09}$	5.00 × $10^{-09}$	5.57 × $10^{-09}$	5.03 × $10^{-09}$	3.73 × $10^{-09}$	2.26 × $10^{-09}$	1.10 × $10^{-09}$	4.21 × $10^{-10}$	1.20 × $10^{-10}$	2.33 × $10^{-11}$	2.68 × $10^{-12}$	1.38 × $10^{-13}$	1.68 × $10^{-15}$	6.33 × $10^{-19}$
13	3.13 × $10^{-12}$	1.99 × $10^{-10}$	1.61 × $10^{-09}$	5.50 × $10^{-09}$	1.13 × $10^{-08}$	1.66 × $10^{-08}$	1.86 × $10^{-08}$	1.67 × $10^{-08}$	1.23 × $10^{-08}$	7.45 × $10^{-09}$	3.69 × $10^{-09}$	1.47 × $10^{-09}$	4.53 × $10^{-10}$	1.03 × $10^{-10}$	1.55 × $10^{-11}$	1.34 × $10^{-12}$	4.87 × $10^{-14}$	3.74 × $10^{-16}$	6.66 × $10^{-20}$
14	1.19 × $10^{-10}$	3.57 × $10^{-09}$	1.83 × $10^{-08}$	4.40 × $10^{-08}$	6.80 × $10^{-08}$	7.73 × $10^{-08}$	6.90 × $10^{-08}$	5.02 × $10^{-08}$	3.01 × $10^{-08}$	1.49 × $10^{-08}$	6.04 × $10^{-09}$	1.96 × $10^{-09}$	4.88 × $10^{-10}$	8.79 × $10^{-11}$	1.04 × $10^{-11}$	6.71 × $10^{-13}$	1.72 × $10^{-14}$	8.30 × $10^{-17}$	7.01 × $10^{-21}$
15	4.52 × $10^{-09}$	6.43 × $10^{-08}$	2.07 × $10^{-07}$	3.52 × $10^{-07}$	4.08 × $10^{-07}$	3.61 × $10^{-07}$	2.56 × $10^{-07}$	1.50 × $10^{-07}$	7.35 × $10^{-08}$	2.98 × $10^{-08}$	9.88 × $10^{-09}$	2.61 × $10^{-09}$	5.25 × $10^{-10}$	7.54 × $10^{-11}$	6.91 × $10^{-12}$	3.36 × $10^{-13}$	6.07 × $10^{-15}$	1.85 × $10^{-17}$	7.38 × $10^{-22}$
16	1.72 × $10^{-07}$	1.16 × $10^{-06}$	2.35 × $10^{-06}$	2.81 × $10^{-06}$	2.45 × $10^{-06}$	1.68 × $10^{-06}$	9.52 × $10^{-07}$	4.51 × $10^{-07}$	1.80 × $10^{-07}$	5.96 × $10^{-08}$	1.62 × $10^{-08}$	3.48 × $10^{-09}$	5.66 × $10^{-10}$	6.46 × $10^{-11}$	4.60 × $10^{-12}$	1.68 × $10^{-13}$	2.14 × $10^{-15}$	4.10 × $10^{-18}$	7.77 × $10^{-23}$
17	6.53 × $10^{-06}$	2.08 × $10^{-05}$	2.66 × $10^{-05}$	2.25 × $10^{-05}$	1.47 × $10^{-05}$	7.85 × $10^{-06}$	3.54 × $10^{-06}$	1.35 × $10^{-06}$	4.39 × $10^{-07}$	1.19 × $10^{-07}$	2.65 × $10^{-08}$	4.64 × $10^{-09}$	6.09 × $10^{-10}$	5.54 × $10^{-11}$	3.07 × $10^{-12}$	8.39 × $10^{-14}$	7.56 × $10^{-16}$	9.11 × $10^{-19}$	8.18 × $10^{-24}$

**Table 10:** Empirical type I error rates of exact and asymptotic methods for K=2.

$n_{1}=n_{2}$	γ	$\pi_{1}=\pi_{2}$	Exact Methods									Asymptotic Methods		
			$p_{L}^{E}$	$p_{\mathrm{SC}}^{E}$	$p_{W}^{E}$	$p_{L}^{M}$	$p_{\mathrm{SC}}^{M}$	$p_{W}^{M}$	$p_{L}^{\mathrm{EM}}$	$p_{\mathrm{SC}}^{\mathrm{EM}}$	$p_{W}^{\mathrm{EM}}$	$p_{L}^{A}$	$p_{\mathrm{SC}}^{A}$	$p_{W}^{A}$
10	0.1	0.3	0.0485	0.0497	0.0514	0.0366	0.0483	0.0404	0.0413	0.0327	0.0419	0.0569	0.0506	0.0855
	0.3		0.0482	0.0511	0.0528	0.0358	0.0491	0.0334	0.0405	0.0351	0.0425	0.0584	0.0514	0.0752
	0.5		0.0528	0.0544	0.0586	0.0428	0.0478	0.0267	0.0455	0.0394	0.0464	0.0686	0.0502	0.0632
	0.7		0.0580	0.0523	0.0546	0.0435	0.0354	0.0156	0.0481	0.0365	0.0414	0.0765	0.0383	0.0427
	0.8		0.0434	0.0371	0.0366	0.0290	0.0204	0.0073	0.0341	0.0243	0.0266	0.0582	0.0224	0.0256
	0.9		0.0149	0.0125	0.0106	0.0079	0.0048	0.0011	0.0105	0.0071	0.0072	0.0203	0.0055	0.0069
	0.1	0.5	0.0404	0.0425	0.0457	0.0340	0.0371	0.0354	0.0358	0.0332	0.0381	0.0485	0.0383	0.0749
	0.3		0.0445	0.0481	0.0559	0.0363	0.0379	0.0341	0.0383	0.0357	0.0431	0.0600	0.0389	0.0726
	0.5		0.0553	0.0550	0.0649	0.0399	0.0359	0.0301	0.0449	0.0371	0.0476	0.0751	0.0380	0.0694
	0.7		0.0549	0.0473	0.0542	0.0316	0.0222	0.0170	0.0404	0.0282	0.0372	0.0716	0.0243	0.0525
	0.8		0.0358	0.0292	0.0331	0.0177	0.0106	0.0072	0.0246	0.0157	0.0212	0.0469	0.0116	0.0314
	0.9		0.0099	0.0078	0.0086	0.0040	0.0019	0.0010	0.0062	0.0036	0.0050	0.0132	0.0021	0.0080
	0.1	0.6	0.0433	0.0451	0.0466	0.0334	0.0404	0.0366	0.0367	0.0327	0.0385	0.0506	0.0423	0.0783
	0.3		0.0458	0.0494	0.0537	0.0365	0.0414	0.0342	0.0387	0.0362	0.0427	0.0584	0.0426	0.0732
	0.5		0.0542	0.0556	0.0618	0.0420	0.0398	0.0295	0.0449	0.0387	0.0471	0.0727	0.0420	0.0671
	0.7		0.0547	0.0495	0.0528	0.0362	0.0261	0.0168	0.0418	0.0316	0.0379	0.0727	0.0285	0.0497
	0.8		0.0370	0.0319	0.0330	0.0214	0.0133	0.0074	0.0262	0.0186	0.0222	0.0496	0.0146	0.0300
	0.9		0.0109	0.0092	0.0088	0.0051	0.0026	0.0010	0.0068	0.0046	0.0054	0.0149	0.0029	0.0078
25	0.1	0.3	0.0493	0.0493	0.0493	0.0358	0.0358	0.0358	0.0409	0.0416	0.0393	0.0527	0.0516	0.0516
	0.3		0.0496	0.0496	0.0496	0.0344	0.0344	0.0344	0.0417	0.0430	0.0399	0.0523	0.0502	0.0501
	0.5		0.0494	0.0494	0.0494	0.0353	0.0353	0.0353	0.0408	0.0424	0.0395	0.0546	0.0514	0.0507
	0.7		0.0484	0.0483	0.0483	0.0400	0.0400	0.0400	0.0408	0.0442	0.0407	0.0599	0.0511	0.0477
	0.8		0.0562	0.0556	0.0556	0.0478	0.0478	0.0478	0.0483	0.0488	0.0440	0.0726	0.0481	0.0431
	0.9		0.0503	0.0497	0.0497	0.0370	0.0370	0.0370	0.0411	0.0378	0.0277	0.0702	0.0259	0.0207
	0.1	0.5	0.0495	0.0495	0.0495	0.0303	0.0303	0.0303	0.0389	0.0398	0.0358	0.0502	0.0477	0.0477
	0.3		0.0484	0.0484	0.0484	0.0312	0.0312	0.0312	0.0399	0.0412	0.0376	0.0496	0.0477	0.0477
	0.5		0.0486	0.0486	0.0486	0.0346	0.0346	0.0346	0.0392	0.0409	0.0386	0.0513	0.0492	0.0491
	0.7		0.0521	0.0521	0.0521	0.0401	0.0401	0.0401	0.0419	0.0451	0.0437	0.0612	0.0507	0.0507
	0.8		0.0593	0.0593	0.0593	0.0431	0.0431	0.0431	0.0467	0.0482	0.0487	0.0795	0.0457	0.0455
	0.9		0.0414	0.0414	0.0414	0.0223	0.0223	0.0223	0.0280	0.0276	0.0295	0.0690	0.0188	0.0186
	0.1	0.6	0.0493	0.0493	0.0493	0.0325	0.0325	0.0325	0.0404	0.0417	0.0386	0.0505	0.0481	0.0481
	0.3		0.0492	0.0492	0.0492	0.0328	0.0328	0.0328	0.0404	0.0417	0.0388	0.0509	0.0486	0.0486
	0.5		0.0486	0.0486	0.0486	0.0348	0.0348	0.0348	0.0399	0.0416	0.0392	0.0525	0.0498	0.0497
	0.7		0.0503	0.0502	0.0502	0.0408	0.0408	0.0408	0.0425	0.0448	0.0428	0.0607	0.0502	0.0496
	0.8		0.0584	0.0581	0.0581	0.0463	0.0463	0.0463	0.0495	0.0495	0.0473	0.0769	0.0454	0.0443
	0.9		0.0438	0.0436	0.0436	0.0280	0.0280	0.0280	0.0339	0.0324	0.0290	0.0685	0.0201	0.0187

Note: The type I error rates of robust region (0.04–0.06) are shown in bold.

**Table 11:** Powers of exact and asymptotic methods for K=2 (balanced π conditions).

$\gamma_{1}$	$\gamma_{2}$	$\pi_{1}=\pi_{2}$	Exact Methods									Asymptotic Methods		
			$p_{L}^{E}$	$p_{\mathrm{SC}}^{E}$	$p_{W}^{E}$	$p_{L}^{M}$	$p_{\mathrm{SC}}^{M}$	$p_{W}^{M}$	$p_{L}^{\mathrm{EM}}$	$p_{\mathrm{SC}}^{\mathrm{EM}}$	$p_{W}^{\mathrm{EM}}$	$p_{L}^{A}$	$p_{\mathrm{SC}}^{A}$	$p_{W}^{A}$
0.1	0.5	0.3	0.1403	0.1437	0.1549	0.1268	0.1368	0.1209	0.1323	0.1325	0.1334	0.1719	0.1593	0.1929
	0.6		0.2108	0.2130	0.2281	0.1945	0.2041	0.1747	0.2006	0.2003	0.1996	0.2472	0.2304	0.2641
	0.7		0.3114	0.3106	0.3317	0.2924	0.2990	0.2489	0.2979	0.2956	0.2943	0.3518	0.3276	0.3572
	0.8		0.4509	0.4439	0.4723	0.4301	0.4283	0.3478	0.4336	0.4253	0.4265	0.4932	0.4556	0.4772
	0.9		0.6408	0.6217	0.6570	0.6192	0.5991	0.4761	0.6201	0.5968	0.6076	0.6812	0.6180	0.6308
0.3	0.6		0.1149	0.1187	0.1284	0.1019	0.1072	0.0781	0.1048	0.1042	0.1069	0.1389	0.1217	0.1378
	0.7		0.1852	0.1869	0.2016	0.1683	0.1698	0.1208	0.1709	0.1671	0.1716	0.2173	0.1863	0.2040
	0.8		0.2976	0.2937	0.3151	0.2764	0.2671	0.1859	0.2779	0.2655	0.2750	0.3409	0.2840	0.3029
	0.9		0.4727	0.4555	0.4846	0.4470	0.4132	0.2824	0.4474	0.4142	0.4357	0.5307	0.4261	0.4461
0.5	0.8		0.1614	0.1570	0.1680	0.1456	0.1290	0.0703	0.1463	0.1333	0.1401	0.2000	0.1372	0.1444
	0.9		0.2818	0.2656	0.2828	0.2568	0.2176	0.1181	0.2581	0.2282	0.2423	0.3453	0.2255	0.2342
0.1	0.5	0.5	0.1442	0.1533	0.1684	0.1186	0.1331	0.1142	0.1273	0.1310	0.1367	0.1671	0.1463	0.1852
	0.6		0.2137	0.2265	0.2465	0.1830	0.1986	0.1711	0.1917	0.1967	0.2052	0.2455	0.2144	0.2647
	0.7		0.3115	0.3277	0.3521	0.2761	0.2904	0.2511	0.2843	0.2899	0.3021	0.3532	0.3078	0.3696
	0.8		0.4462	0.4631	0.4894	0.4071	0.4148	0.3607	0.4151	0.4186	0.4350	0.4969	0.4322	0.5015
	0.9		0.6288	0.6382	0.6611	0.5876	0.5786	0.5076	0.5971	0.5922	0.6126	0.6835	0.5927	0.6587
0.3	0.6		0.1124	0.1210	0.1340	0.0946	0.0969	0.0777	0.0991	0.0999	0.1076	0.1383	0.1058	0.1413
	0.7		0.1797	0.1895	0.2065	0.1547	0.1533	0.1233	0.1609	0.1603	0.1717	0.2167	0.1643	0.2113
	0.8		0.2861	0.2942	0.3147	0.2507	0.2400	0.1936	0.2602	0.2554	0.2718	0.3376	0.2529	0.3119
	0.9		0.4501	0.4483	0.4700	0.3996	0.3690	0.2992	0.4153	0.4002	0.4229	0.5181	0.3823	0.4488
0.5	0.8		0.1535	0.1508	0.1627	0.1232	0.1038	0.0754	0.1334	0.1249	0.1361	0.1950	0.1113	0.1476
	0.9		0.2589	0.2471	0.2623	0.2089	0.1717	0.1252	0.2267	0.2100	0.2266	0.3219	0.1813	0.2295
0.1	0.5	0.6	0.1478	0.1549	0.1678	0.1210	0.1378	0.1163	0.1307	0.1341	0.1413	0.1679	0.1531	0.1878
	0.6		0.2183	0.2279	0.2453	0.1865	0.2048	0.1721	0.1960	0.2006	0.2109	0.2458	0.2227	0.2650
	0.7		0.3169	0.3287	0.3504	0.2813	0.2983	0.2500	0.2896	0.2942	0.3088	0.3529	0.3177	0.3672
	0.8		0.4516	0.4633	0.4876	0.4150	0.4244	0.3560	0.4212	0.4223	0.4425	0.4959	0.4430	0.4970
	0.9		0.6323	0.6376	0.6602	0.5992	0.5891	0.4975	0.6040	0.5929	0.6197	0.6827	0.6029	0.6544
0.3	0.6		0.1139	0.1218	0.1337	0.0971	0.1014	0.0771	0.1008	0.1016	0.1114	0.1373	0.1119	0.1407
	0.7		0.1814	0.1905	0.2066	0.1595	0.1599	0.1217	0.1638	0.1626	0.1770	0.2158	0.1726	0.2101
	0.8		0.2881	0.2959	0.3161	0.2598	0.2500	0.1906	0.2653	0.2582	0.2792	0.3377	0.2642	0.3108
	0.9		0.4521	0.4515	0.4741	0.4160	0.3839	0.2940	0.4243	0.4033	0.4329	0.5212	0.3973	0.4495
0.5	0.8		0.1536	0.1529	0.1648	0.1313	0.1116	0.0739	0.1373	0.1275	0.1410	0.1960	0.1199	0.1468
	0.9		0.2608	0.2521	0.2681	0.2248	0.1853	0.1235	0.2357	0.2150	0.2357	0.3281	0.1952	0.2305

**Table 12:** Values of test statistics and *p*-value.

Value	Test Statistics		
	$T_{L}$	$T_{\mathrm{SC}}$	$T_{W}$
Statistic value	5.0377	5.0762	5.1107
*p*-value	0.0248	0.0243	0.0238

**Table 13:** Comparison of asymptotic and exact *p*-value.

Method	A Approach			E Approach			M Approach			E + M Approach		
	$p_{L}^{A}$	$p_{\mathrm{SC}}^{A}$	$p_{W}^{A}$	$p_{L}^{E}$	$p_{\mathrm{SC}}^{E}$	$p_{W}^{E}$	$p_{L}^{M}$	$p_{\mathrm{SC}}^{M}$	$p_{W}^{M}$	$p_{L}^{\mathrm{EM}}$	$p_{\mathrm{SC}}^{\mathrm{EM}}$	$p_{W}^{\mathrm{EM}}$
*p*-value	0.1558	0.1607	0.1492	0.1953	0.1952	0.0854	0.2194	0.2076	0.2039	0.1989	0.1999	0.2127

## Data Availability

Clinical data referred to are from Hannah et al. [16] and Hang et al. [20].

## Abbreviations

The following abbreviations are used in this manuscript:

MLEs:	maximum likelihood estimates.	$T_{L}$:	likelihood ratio statistic.
$T_{SC}$:	score statistic.	$T_{W}$:	Wald-type statistic.
A:	asymptotic approach.	E:	E approach.
M:	M approach.	E+M:	E+M approach.
PVR:	proliferative vitreoretinopathy.	

## Appendix A

### Appendix A.1. The Fisher Information Matrix of MLEs under H_0_

Let $I_{1}$ be the (K+1)×(K+1) Fisher information matrix, and

$$\begin{array}{ccc} I_{1}=\left[\begin{array}{cc} I_{111} & I_{112}\\ I_{112}^{T} & I_{122} \end{array}\right] & = & \left[\begin{array}{cccc} \;E\left(-\frac{\partial^{2}l_{0}}{\partial \gamma^{2}}\right) & E\left(-\frac{\partial^{2}l_{0}}{\partial \gamma \partial \pi_{1}}\right) & \cdots & E\left(-\frac{\partial^{2}l_{0}}{\partial \gamma \partial \pi_{K}}\right)\\ E\left(-\frac{\partial^{2}l_{0}}{\partial \gamma \partial \pi_{1}}\right) & E(\;-\frac{\partial^{2}l_{0}}{\partial \pi_{1}^{2}}\;) & \cdots & \vdots \\ \vdots & \vdots & \ddots & \vdots \\ E\left(-\frac{\partial^{2}l_{0}}{\partial \gamma \partial \pi_{K}}\right) & \cdots & \cdots & E(\;-\frac{\partial^{2}l_{0}}{\partial \pi_{K}^{2}}\;) \end{array}\right]\\ & = & \left[\begin{array}{cccc} \;\sum_{k=1}^{K}\frac{n_{k}}{4}a_{k}^{2}b_{k} & \frac{n_{1}a_{1}c_{1}}{2} & \cdots & \frac{n_{K}a_{K}c_{K}}{2}\\ \frac{n_{1}a_{1}c_{1}}{2} & n_{1}d_{1} & \cdots & \vdots \\ \vdots & \vdots & \ddots & \vdots \\ \frac{n_{K}a_{K}c_{K}}{2} & \cdots & \cdots & n_{K}d_{K} \end{array}\right], \end{array}$$

where

$$\begin{array}{c} \begin{array}{cc} a_{k} & =1-2\pi_{k}\left(1-\pi_{k}\right),\\ b_{k} & =\frac{1}{P_{1k}\left(\gamma ,\pi_{k}\right)}+\frac{4}{P_{2k}\left(\gamma ,\pi_{k}\right)}+\frac{1}{P_{3k}\left(\gamma ,\pi_{k}\right)},\\ c_{k} & =\frac{1}{P_{1k}\left(\gamma ,\pi_{k}\right)}-\frac{1}{P_{3k}\left(\gamma ,\pi_{k}\right)}+\left(1-\gamma \right)\left(1-2\pi_{k}\right)b_{k},\\ d_{k} & =\frac{1}{P_{1k}\left(\gamma ,\pi_{k}\right)}+\frac{1}{P_{3k}\left(\gamma ,\pi_{k}\right)}+\left(1-\gamma \right)\left(1-2\pi_{k}\right)\times \left(\frac{1}{P_{1k}\left(\gamma ,\pi_{k}\right)}-\frac{1}{P_{3k}\left(\gamma ,\pi_{k}\right)}+c_{k}\right) \end{array} \end{array}$$

for k=1,2,…,K.

### Appendix A.2. The Fisher Information Matrix I_2_ of the Score Statistic

For the observed data $N=(n_{1},n_{2},\ldots ,n_{K})$, the Fisher information matrix of score statistic is

$$\begin{array}{c} I_{2}=\left[\begin{array}{cc} I_{211} & I_{212}\\ I_{221} & I_{222} \end{array}\right]=\left[\begin{array}{cccccc} E(\;-\frac{\partial^{2}l_{1}}{\partial \gamma_{1}^{2}}\;) & \cdots & \cdots & E(\;-\frac{\partial^{2}l_{1}}{\partial \gamma_{1}\partial \pi_{1}}\;) & \cdots & \cdots \\ \vdots & \ddots & \vdots & \vdots & \ddots & \vdots \\ \cdots & \cdots & E(\;-\frac{\partial^{2}l_{K}}{\partial \gamma_{K}^{2}}\;) & \cdots & \cdots & E(\;-\frac{\partial^{2}l_{K}}{\partial \gamma_{K}\partial \pi_{K}}\;)\\ E(\;-\frac{\partial^{2}l_{1}}{\partial \gamma_{1}\partial \pi_{1}}\;) & \cdots & \cdots & E(\;-\frac{\partial^{2}l_{1}}{\partial \pi_{1}^{2}}\;) & \cdots & \cdots \\ \vdots & \ddots & \vdots & \vdots & \ddots & \vdots \\ \cdots & \cdots & E(\;-\frac{\partial^{2}l_{K}}{\partial \gamma_{K}\partial \pi_{K}}\;) & \cdots & \cdots & E(\;-\frac{\partial^{2}l_{K}}{\partial \pi_{K}^{2}}\;) \end{array}\right], \end{array}$$

Since $E\left(n_{lk}\right)=n_{k}P_{lk}\left(\gamma_{k},\pi_{k}\right)\;\left(l=1,2,3\right)$, we can obtain $E\left(\;-\frac{\partial^{2}l_{k}}{\partial \gamma_{k}^{2}}\;\right)=\frac{n_{k}a_{k}^{2}b_{k}}{4}$, $E\left(\;-\frac{\partial^{2}l_{k}}{\partial \gamma_{k}\partial \pi_{k}}\;\right)=\frac{n_{k}a_{k}c_{k}}{2},$ and $E\left(\;-\frac{\partial^{2}l_{k}}{\partial \pi_{k}^{2}}\;\right)=n_{k}d_{k}.$ Thus, the Fisher information matrix $I_{2}$ is simplified as

$$\begin{array}{c} I_{2}=\frac{1}{4}\left[\begin{array}{cccccc} n_{1}a_{1}^{2}b_{1} & \cdots & \cdots & 2n_{1}a_{1}c_{1} & \cdots & \cdots \\ \vdots & \ddots & \vdots & \vdots & \ddots & \vdots \\ \cdots & \cdots & n_{K}a_{K}^{2}b_{K} & \cdots & \cdots & 2n_{K}a_{K}c_{K}\\ 2n_{1}a_{1}c_{1} & \cdots & \cdots & 4n_{1}d_{1} & \cdots & \cdots \\ \vdots & \ddots & \vdots & \vdots & \ddots & \vdots \\ \cdots & \cdots & 2n_{K}a_{K}c_{K} & \cdots & \cdots & 4n_{K}d_{K} \end{array}\right]. \end{array}$$

### Appendix A.3. The Simplification of T_W_

To obtain the simplification of $T_{W}$, we calculate $CI_{3}^{-1}C^{T}$ and obtain

$$\begin{array}{c} CI_{3}^{-1}C^{T}=\left[\begin{array}{ccccccc} g_{{}_{1}}+g_{{}_{2}} & -g_{{}_{2}} & 0 & 0 & \cdots & \cdots & 0\\ -g_{{}_{2}} & g_{{}_{2}}+g_{{}_{3}} & -g_{{}_{3}} & 0 & \cdots & \cdots & 0\\ \vdots & \vdots & \vdots & \vdots & \vdots & \vdots & \vdots \\ 0 & 0 & \cdots & \cdots & -g_{{}_{K-2}} & g_{{}_{K-2}}+g_{{}_{K-1}} & -g_{{}_{K-1}}\\ 0 & 0 & \cdots & \cdots & 0 & -g_{{}_{K-1}} & g_{{}_{K-1}}+g_{{}_{K}} \end{array}\right], \end{array}$$

where $g_{{}_{k}}=\frac{4d_{k}}{n_{{}_{k}}a_{k}^{2}\left(b_{k}d_{k}-c_{k}^{2}\right)}$. Obviously, ${\left(CI_{3}^{-1}C^{T}\right)}^{-1}$ is a symmetric matrix, in which

$$\begin{array}{c} \left\{\begin{array}{cc} {\left(CI_{3}^{-1}C^{T}\right)}_{i,j}^{-1} & =\;g_{{}_{i+1}}\cdots g_{{}_{j}}\frac{h_{{}_{j+1}}h_{{}_{K-1}}}{s_{{}_{i}}\cdots s_{{}_{K-1}}},\;\forall j>i,\\ {\left(CI_{3}^{-1}C^{T}\right)}_{i,i}^{-1} & =\;\frac{h_{{}_{i+1}}h_{{}_{K-1}}}{s_{i}\cdots s_{{}_{K-1}}},\;\;\;\;\forall i, \end{array}\right. \end{array}$$

where

$$\begin{array}{c} \left\{\begin{array}{c} \;h_{{}_{K}}=1,h_{{}_{K-1}}=g_{{}_{K-1}}+g_{{}_{K}},\\ h_{{}_{i}}=\left(g_{{}_{i}}+g_{{}_{i+1}}-\frac{g_{{}_{i+1}}^{2}}{h_{{}_{i+1}}}\right),\;i=1,2,\ldots ,K-2,\\ s_{{}_{1}}=g_{{}_{1}}+g_{{}_{2}},s_{{}_{i}}=\left(g_{{}_{i}}+g_{{}_{i+1}}\right)-\frac{g_{{}_{i}}^{2}}{s_{{}_{i-1}}},\;i=2,\ldots ,K-1. \end{array}\right. \end{array}$$

By computation, the simplified form of $T_{W}$ is given as

$$T_{W}=\sum_{i=1}^{K-1}\sum_{j=1}^{K-1}\left({\hat{\gamma }}_{i}-{\hat{\gamma }}_{i+1}\right)\left({\hat{\gamma }}_{j}-{\hat{\gamma }}_{j+1}\right){\left(CI_{3}^{-1}C^{T}\right)}_{i,j}^{-1}.$$

### Appendix A.4. Other Tables and Figures

**Table A1:** Empirical type I error rates of asymptotic statistics for K=3 (balanced sample sizes).

Balanced π Conditions						Unbalanced π Conditions							
$n_{1}=n_{2}=n_{3}$	γ	$\pi_{1}=\pi_{2}=\pi_{3}$	$T_{L}$	$T_{\mathrm{SC}}$	$T_{W}$	$n_{1}=n_{2}=n_{3}$	γ	$\pi_{1}$	$\pi_{2}$	$\pi_{3}$	$T_{L}$	$T_{\mathrm{SC}}$	$T_{W}$
10	0.1	0.3	0.0527	0.0454	0.1192	10	0.1	0.3	0.5	0.5	0.0515	0.0420	0.1030
	0.3		0.0576	0.0462	0.1069		0.3				0.0510	0.0393	0.0958
	0.5		0.0421	0.0337	0.0655		0.5				0.0338	0.0259	0.0627
	0.7		0.0152	0.0138	0.0167		0.7				0.0124	0.0114	0.0147
	0.9		0.0014	0.0014	0.0000		0.9				0.0005	0.0007	0.0001
	0.1	0.6	0.0512	0.0443	0.1015		0.1	0.6	0.4	0.2	0.0525	0.0439	0.1115
	0.3		0.0444	0.0341	0.0890		0.3				0.0529	0.0406	0.0974
	0.5		0.0307	0.0241	0.0574		0.5				0.0412	0.0315	0.0671
	0.7		0.0129	0.0118	0.0157		0.7				0.0161	0.0144	0.0204
	0.9		0.0006	0.0008	0.0001		0.9				0.0005	0.0008	0.0000
	0.1	0.5	0.0508	0.0413	0.0990		0.1	0.5	0.4	0.3	0.0578	0.0472	0.1078
	0.3		0.0437	0.0317	0.0857		0.3				0.0524	0.0416	0.0992
	0.5		0.0313	0.0223	0.0574		0.5				0.0379	0.0290	0.0662
	0.7		0.0086	0.0082	0.0127		0.7				0.0122	0.0115	0.0145
	0.9		0.0002	0.0003	0.0002		0.9				0.0004	0.0004	0.0001
50	0.1	0.3	0.0547	0.0528	0.0681	50	0.1	0.3	0.5	0.5	0.0494	0.0480	0.0600
	0.3		0.0498	0.0482	0.0600		0.3				0.0507	0.0477	0.0602
	0.5		0.0533	0.0514	0.0632		0.5				0.0513	0.0471	0.0586
	0.7		0.0548	0.0495	0.0606		0.7				0.0537	0.0479	0.0634
	0.9		0.0338	0.0289	0.0266		0.9				0.0272	0.0242	0.0221
	0.1	0.6	0.0480	0.0464	0.0583		0.1	0.6	0.4	0.2	0.0655	0.0646	0.0823
	0.3		0.0470	0.0450	0.0563		0.3				0.0563	0.0541	0.0671
	0.5		0.0517	0.0489	0.0590		0.5				0.0510	0.0480	0.0600
	0.7		0.0528	0.0457	0.0613		0.7				0.0577	0.0505	0.0628
	0.9		0.0237	0.0216	0.0211		0.9				0.0318	0.0286	0.0249
	0.1	0.5	0.0502	0.0476	0.0588		0.1	0.5	0.4	0.3	0.0485	0.0468	0.0598
	0.3		0.0483	0.0455	0.0586		0.3				0.0528	0.0499	0.0627
	0.5		0.0514	0.0477	0.0622		0.5				0.0531	0.0489	0.0601
	0.7		0.0526	0.0456	0.0588		0.7				0.0538	0.0470	0.0609
	0.9		0.0229	0.0220	0.0236		0.9				0.0281	0.0269	0.0248
100	0.1	0.3	0.0520	0.0515	0.0588	100	0.1	0.3	0.5	0.5	0.0509	0.0506	0.0562
	0.3		0.0518	0.0507	0.0571		0.3				0.0501	0.0491	0.0545
	0.5		0.0487	0.0480	0.0530		0.5				0.0495	0.0470	0.0531
	0.7		0.0523	0.0503	0.0542		0.7				0.0481	0.0455	0.0550
	0.9		0.0563	0.0473	0.0605		0.9				0.0524	0.0402	0.0618
	0.1	0.6	0.0492	0.0489	0.0543		0.1	0.6	0.4	0.2	0.0774	0.0772	0.0877
	0.3		0.0500	0.0484	0.0551		0.3				0.0542	0.0528	0.0619
	0.5		0.0579	0.0559	0.0631		0.5				0.0520	0.0509	0.0565
	0.7		0.0514	0.0499	0.0569		0.7				0.0529	0.0513	0.0539
	0.9		0.0529	0.0431	0.0610		0.9				0.0529	0.0430	0.0578
	0.1	0.5	0.0499	0.0487	0.0545		0.1	0.5	0.4	0.3	0.0497	0.0479	0.0546
	0.3		0.0475	0.0469	0.0519		0.3				0.0466	0.0461	0.0511
	0.5		0.0518	0.0496	0.0555		0.5				0.0478	0.0470	0.0513
	0.7		0.0525	0.0496	0.0581		0.7				0.0506	0.0478	0.0568
	0.9		0.0563	0.0428	0.0653		0.9				0.0555	0.0471	0.0617

Note: The type I error rates of robust region (0.04–0.06) are shown in bold.

**Table A2:** Empirical type I error rates of homogeneity tests for K=4 (balanced sample sizes).

Balanced π Conditions						Unbalanced π Conditions								
$n_{1}=n_{2}=n_{3}=n_{4}$	γ	$\pi_{1}=\pi_{2}=\pi_{3}=\pi_{4}$	$T_{L}$	$T_{\mathrm{SC}}$	$T_{W}$	$n_{1}=n_{2}=n_{3}=n_{4}$	γ	$\pi_{1}$	$\pi_{2}$	$\pi_{3}$	$\pi_{4}$	$T_{L}$	$T_{\mathrm{SC}}$	$T_{W}$
10	0.1	0.3	0.0559	0.0437	0.1519	10	0.1	0.3	0.5	0.5	0.2	0.0541	0.0429	0.1287
	0.3		0.0595	0.0465	0.1349		0.3					0.0466	0.0349	0.1166
	0.5		0.0408	0.0325	0.0857		0.5					0.0353	0.0277	0.0763
	0.7		0.0126	0.0137	0.0159		0.7					0.0104	0.0114	0.0147
	0.9		0.0005	0.0015	0.0001		0.9					0.0005	0.0016	0.0001
	0.1	0.6	0.0543	0.0455	0.1281		0.1	0.6	0.4	0.2	0.4	0.0594	0.0482	0.1387
	0.3		0.0462	0.0345	0.1206		0.3					0.0495	0.0376	0.1175
	0.5		0.0265	0.0204	0.0671		0.5					0.0375	0.0290	0.0783
	0.7		0.0084	0.0096	0.0120		0.7					0.0108	0.0122	0.0150
	0.9		0.0002	0.0006	0.0001		0.9					0.0001	0.0005	0.0000
	0.1	0.5	0.0491	0.0404	0.1158		0.1	0.5	0.4	0.3	0.3	0.0583	0.0475	0.1433
	0.3		0.0436	0.0340	0.1049		0.3					0.0533	0.0396	0.1233
	0.5		0.0234	0.0193	0.0572		0.5					0.0328	0.0250	0.0768
	0.7		0.0073	0.0093	0.0109		0.7					0.0095	0.0112	0.0145
	0.9		0.0002	0.0008	0.0000		0.9					0.0005	0.0011	0.0002
50	0.1	0.3	0.0531	0.0515	0.0694	50	0.1	0.3	0.5	0.5	0.2	0.0638	0.0617	0.0831
	0.3		0.0504	0.0485	0.0659		0.3					0.0525	0.0501	0.0671
	0.5		0.0532	0.0499	0.0691		0.5					0.0508	0.0472	0.0633
	0.7		0.0577	0.0497	0.0682		0.7					0.0537	0.0466	0.0705
	0.9		0.0304	0.0301	0.0256		0.9					0.0264	0.0265	0.0239
	0.1	0.6	0.0511	0.0477	0.0662		0.1	0.6	0.4	0.2	0.4	0.0621	0.0608	0.0810
	0.3		0.0525	0.0495	0.0660		0.3					0.0523	0.0497	0.0692
	0.5		0.0566	0.0513	0.0688		0.5					0.0525	0.0485	0.0658
	0.7		0.0549	0.0475	0.0726		0.7					0.0532	0.0457	0.0674
	0.9		0.0191	0.0208	0.0185		0.9					0.0263	0.0260	0.0236
	0.1	0.5	0.0433	0.0416	0.0578		0.1	0.5	0.4	0.3	0.3	0.0486	0.0461	0.0633
	0.3		0.0477	0.0444	0.0593		0.3					0.0499	0.0473	0.0651
	0.5		0.0502	0.0458	0.0601		0.5					0.0519	0.0472	0.0631
	0.7		0.0549	0.0452	0.0739		0.7					0.0549	0.0476	0.0688
	0.9		0.0223	0.0244	0.0206		0.9					0.0287	0.0288	0.0256
100	0.1	0.3	0.0508	0.0499	0.0585	100	0.1	0.3	0.5	0.5	0.2	0.0805	0.0794	0.0921
	0.3		0.0494	0.0481	0.0571		0.3					0.0551	0.0551	0.0631
	0.5		0.0498	0.0485	0.0568		0.5					0.0471	0.0457	0.0531
	0.7		0.0504	0.0488	0.0556		0.7					0.0531	0.0505	0.0595
	0.9		0.0568	0.0464	0.0712		0.9					0.0513	0.0408	0.0743
	0.1	0.6	0.0462	0.0454	0.0523		0.1	0.6	0.4	0.2	0.4	0.0794	0.0789	0.0960
	0.3		0.0484	0.0473	0.0547		0.3					0.0551	0.0535	0.0647
	0.5		0.0493	0.0479	0.0556		0.5					0.0498	0.0473	0.0566
	0.7		0.0498	0.0472	0.0599		0.7					0.0484	0.0465	0.0548
	0.9		0.0494	0.0401	0.0737		0.9					0.0586	0.0482	0.0804
	0.1	0.5	0.0476	0.0464	0.0531		0.1	0.5	0.4	0.3	0.3	0.0499	0.0491	0.0579
	0.3		0.0493	0.0469	0.0561		0.3					0.0499	0.0486	0.0582
	0.5		0.0484	0.0459	0.0571		0.5					0.0495	0.0476	0.0577
	0.7		0.0536	0.0471	0.0620		0.7					0.0523	0.0493	0.0584
	0.9		0.0482	0.0407	0.0717		0.9					0.0587	0.0467	0.0783

Note: The type I error rates of robust region (0.04–0.06) are shown in bold.

**Table A3:** Powers of exact and asymptotic methods for K=2 (unbalanced π conditions).

$\gamma_{1}$	$\gamma_{2}$	$\pi_{1}$	$\pi_{2}$	Exact Methods									Asymptotic Methods		
				$p_{L}^{E}$	$p_{\mathrm{SC}}^{E}$	$p_{W}^{E}$	$p_{L}^{M}$	$p_{\mathrm{SC}}^{M}$	$p_{W}^{M}$	$p_{L}^{\mathrm{EM}}$	$p_{\mathrm{SC}}^{\mathrm{EM}}$	$p_{W}^{\mathrm{EM}}$	$p_{L}^{A}$	$p_{\mathrm{SC}}^{A}$	$p_{W}^{A}$
0.1	0.5	0.3	0.5	0.1668	0.1656	0.1769	0.1466	0.1618	0.1341	0.1523	0.1383	0.1565	0.1808	0.1679	0.1945
	0.6			0.2400	0.2374	0.2520	0.2156	0.2318	0.1894	0.2208	0.2032	0.2263	0.2579	0.2386	0.2660
	0.7			0.3394	0.3341	0.3526	0.3110	0.3254	0.2629	0.3151	0.2937	0.3217	0.3619	0.3327	0.3599
	0.8			0.4720	0.4612	0.4844	0.4408	0.4469	0.3585	0.4431	0.4169	0.4493	0.4998	0.4540	0.4807
	0.9			0.6459	0.6253	0.6541	0.6146	0.6008	0.4816	0.6152	0.5807	0.6167	0.6807	0.6061	0.6341
0.3	0.6			0.1310	0.1310	0.1429	0.1112	0.1202	0.0850	0.1141	0.1038	0.1207	0.1496	0.1240	0.1483
	0.7			0.2027	0.2001	0.2161	0.1776	0.1819	0.1292	0.1799	0.1627	0.1879	0.2297	0.1862	0.2181
	0.8			0.3110	0.3030	0.3242	0.2806	0.2722	0.1946	0.2821	0.2528	0.2899	0.3508	0.2766	0.3186
	0.9			0.4707	0.4519	0.4792	0.4366	0.4001	0.2890	0.4374	0.3868	0.4402	0.5298	0.4038	0.4584
0.5	0.8			0.1636	0.1564	0.1692	0.1421	0.1228	0.0764	0.1440	0.1208	0.1462	0.2086	0.1255	0.1583
	0.9			0.2690	0.2518	0.2703	0.2378	0.1958	0.1233	0.2406	0.1987	0.2400	0.3427	0.1987	0.2456
0.1	0.5	0.5	0.4	0.1399	0.1501	0.1629	0.1143	0.1382	0.1110	0.1236	0.1209	0.1335	0.1612	0.1455	0.1823
	0.6			0.2089	0.2231	0.2406	0.1782	0.2056	0.1668	0.1875	0.1845	0.2013	0.2386	0.2140	0.2604
	0.7			0.3068	0.3248	0.3471	0.2718	0.2992	0.2462	0.2803	0.2763	0.2978	0.3462	0.3086	0.3645
	0.8			0.4428	0.4619	0.4868	0.4049	0.4249	0.3558	0.4125	0.4056	0.4312	0.4910	0.4349	0.4967
	0.9			0.6287	0.6403	0.6624	0.5899	0.5886	0.5042	0.5980	0.5836	0.6109	0.6803	0.5983	0.6557
0.3	0.6			0.1097	0.1194	0.1309	0.0927	0.1015	0.0747	0.0967	0.0943	0.1049	0.1332	0.1069	0.1373
	0.7			0.1768	0.1882	0.2036	0.1533	0.1594	0.1194	0.1585	0.1535	0.1682	0.2106	0.1664	0.2062
	0.8			0.2845	0.2947	0.3137	0.2517	0.2481	0.1895	0.2593	0.2485	0.2685	0.3316	0.2573	0.3066
	0.9			0.4527	0.4532	0.4734	0.4062	0.3795	0.2959	0.4189	0.3961	0.4218	0.5144	0.3908	0.4450
0.5	0.8			0.1542	0.1532	0.1633	0.1268	0.1094	0.0732	0.1348	0.1227	0.1344	0.1908	0.1160	0.1438
	0.9			0.2642	0.2540	0.2668	0.2182	0.1804	0.1232	0.2326	0.2092	0.2269	0.3195	0.1902	0.2265
0.1	0.5	0.6	0.4	0.1478	0.1552	0.1678	0.1210	0.1453	0.1163	0.1307	0.1255	0.1391	0.1679	0.1531	0.1878
	0.6			0.2184	0.2283	0.2453	0.1865	0.2141	0.1721	0.1961	0.1894	0.2076	0.2458	0.2227	0.2650
	0.7			0.3170	0.3294	0.3504	0.2813	0.3085	0.2500	0.2896	0.2807	0.3041	0.3529	0.3177	0.3672
	0.8			0.4517	0.4645	0.4876	0.4150	0.4338	0.3560	0.4212	0.4078	0.4364	0.4959	0.4430	0.4970
	0.9			0.6325	0.6398	0.6602	0.5992	0.5950	0.4975	0.6040	0.5808	0.6130	0.6827	0.6029	0.6544
0.3	0.6			0.1140	0.1225	0.1337	0.0971	0.1065	0.0771	0.1008	0.0969	0.1079	0.1373	0.1119	0.1407
	0.7			0.1816	0.1918	0.2066	0.1595	0.1659	0.1217	0.1639	0.1562	0.1718	0.2158	0.1726	0.2101
	0.8			0.2884	0.2982	0.3161	0.2598	0.2562	0.1906	0.2654	0.2502	0.2719	0.3377	0.2642	0.3108
	0.9			0.4527	0.4555	0.4741	0.4160	0.3885	0.2940	0.4245	0.3948	0.4236	0.5212	0.3973	0.4495
0.5	0.8			0.1541	0.1556	0.1648	0.1313	0.1142	0.0739	0.1374	0.1234	0.1349	0.1960	0.1199	0.1468
	0.9			0.2618	0.2568	0.2681	0.2248	0.1873	0.1235	0.2360	0.2089	0.2266	0.3281	0.1952	0.2305

**Table A4:** Powers of exact and asymptotic methods for K=3 (balanced π conditions).

$\gamma_{1}=\gamma_{3}$	$\gamma_{2}$	$\pi_{1}=\pi_{2}=\pi_{3}$	Exact Methods									Asymptotic Methods		
			$p_{L}^{E}$	$p_{\mathrm{SC}}^{E}$	$p_{W}^{E}$	$p_{L}^{M}$	$p_{\mathrm{SC}}^{M}$	$p_{W}^{M}$	$p_{L}^{\mathrm{EM}}$	$p_{\mathrm{SC}}^{\mathrm{EM}}$	$p_{W}^{\mathrm{EM}}$	$p_{L}^{A}$	$p_{\mathrm{SC}}^{A}$	$p_{W}^{A}$
0.1	0.5	0.3	0.1351	0.1389	0.1421	0.1049	0.0929	0.1089	0.1216	0.1274	0.1150	0.1596	0.1318	0.2366
	0.6		0.1983	0.2002	0.2089	0.1606	0.1383	0.1583	0.1803	0.1845	0.1735	0.2316	0.1901	0.3167
	0.7		0.2905	0.2860	0.3075	0.2460	0.2047	0.2328	0.2677	0.2661	0.2635	0.3342	0.2729	0.4258
	0.8		0.4222	0.4028	0.4504	0.3737	0.2991	0.3433	0.3959	0.3799	0.3994	0.4762	0.3872	0.5682
	0.9		0.6067	0.5572	0.6533	0.5607	0.4296	0.5051	0.5807	0.5349	0.6011	0.6684	0.5407	0.7462
0.3	0.6		0.1055	0.1044	0.1183	0.0862	0.0662	0.0686	0.0951	0.0959	0.0949	0.1328	0.0967	0.1829
	0.7		0.1642	0.1548	0.1858	0.1391	0.1008	0.1081	0.1499	0.1436	0.1534	0.2035	0.1439	0.2632
	0.8		0.2602	0.2319	0.2989	0.2275	0.1543	0.1744	0.2404	0.2174	0.2531	0.3171	0.2166	0.3880
	0.9		0.4124	0.3459	0.4827	0.3707	0.2345	0.2822	0.3859	0.3282	0.4177	0.4943	0.3252	0.5742
0.5	0.8		0.1351	0.1169	0.1550	0.1087	0.0666	0.0565	0.1205	0.1085	0.1177	0.1799	0.1006	0.2119
	0.9		0.2198	0.1787	0.2595	0.1785	0.1027	0.0951	0.1970	0.1670	0.1991	0.2897	0.1538	0.3369
0.1	0.5	0.5	0.1241	0.1273	0.1355	0.1002	0.0855	0.1008	0.1131	0.1186	0.1119	0.1470	0.1146	0.2295
	0.6		0.1851	0.1847	0.2035	0.1549	0.1270	0.1544	0.1710	0.1735	0.1729	0.2164	0.1675	0.3148
	0.7		0.2748	0.2656	0.3035	0.2377	0.1864	0.2352	0.2573	0.2515	0.2655	0.3166	0.2430	0.4281
	0.8		0.4032	0.3762	0.4466	0.3600	0.2691	0.3535	0.3822	0.3590	0.4022	0.4569	0.3476	0.5725
	0.9		0.5829	0.5231	0.6462	0.5366	0.3809	0.5230	0.5588	0.5033	0.5995	0.6483	0.4888	0.7493
0.3	0.6		0.1004	0.0956	0.1207	0.0808	0.0535	0.0723	0.0904	0.0878	0.0981	0.1298	0.0808	0.1884
	0.7		0.1546	0.1408	0.1877	0.1274	0.0800	0.1138	0.1407	0.1302	0.1556	0.1969	0.1202	0.2698
	0.8		0.2406	0.2083	0.2960	0.2026	0.1198	0.1810	0.2210	0.1938	0.2498	0.3026	0.1798	0.3930
	0.9		0.3727	0.3057	0.4650	0.3201	0.1777	0.2861	0.3451	0.2866	0.3993	0.4634	0.2675	0.5732
0.5	0.8		0.1185	0.1056	0.1532	0.0862	0.0481	0.0577	0.1051	0.0954	0.1079	0.1634	0.0801	0.2225
	0.9		0.1841	0.1566	0.2393	0.1352	0.0722	0.0910	0.1643	0.1426	0.1710	0.2517	0.1202	0.3353
0.1	0.5	0.6	0.1278	0.1307	0.1389	0.1021	0.0892	0.1028	0.1156	0.1214	0.1128	0.1502	0.1200	0.2328
	0.6		0.1895	0.1891	0.2066	0.1580	0.1329	0.1560	0.1743	0.1771	0.1738	0.2201	0.1746	0.3180
	0.7		0.2802	0.2713	0.3063	0.2427	0.1958	0.2361	0.2615	0.2565	0.2664	0.3208	0.2523	0.4312
	0.8		0.4101	0.3837	0.4492	0.3678	0.2835	0.3534	0.3882	0.3662	0.4036	0.4616	0.3598	0.5753
	0.9		0.5917	0.5329	0.6489	0.5486	0.4025	0.5213	0.5676	0.5136	0.6019	0.6536	0.5043	0.7510
0.3	0.6		0.1023	0.0984	0.1197	0.0833	0.0583	0.0711	0.0922	0.0903	0.0973	0.1300	0.0853	0.1879
	0.7		0.1583	0.1452	0.1868	0.1324	0.0876	0.1126	0.1442	0.1345	0.1556	0.1983	0.1269	0.2695
	0.8		0.2478	0.2156	0.2964	0.2123	0.1320	0.1804	0.2280	0.2014	0.2523	0.3063	0.1901	0.3933
	0.9		0.3865	0.3180	0.4692	0.3381	0.1970	0.2873	0.3585	0.2995	0.4069	0.4721	0.2831	0.5745
0.5	0.8		0.1247	0.1088	0.1537	0.0936	0.0542	0.0580	0.1104	0.0996	0.1125	0.1677	0.0850	0.2203
	0.9		0.1958	0.1627	0.2446	0.1484	0.0819	0.0932	0.1744	0.1499	0.1813	0.2616	0.1280	0.3366

**Table A5:** Powers of exact and asymptotic methods for K=3 (unbalanced π conditions).

$\gamma_{1}=\gamma_{3}$	$\gamma_{2}$	$\pi_{1}$	$\pi_{2}$	$\pi_{3}$	Exact Methods									Asymptotic Methods		
					$p_{L}^{E}$	$p_{\mathrm{SC}}^{E}$	$p_{W}^{E}$	$p_{L}^{M}$	$p_{\mathrm{SC}}^{M}$	$p_{W}^{M}$	$p_{L}^{\mathrm{EM}}$	$p_{\mathrm{SC}}^{\mathrm{EM}}$	$p_{W}^{\mathrm{EM}}$	$p_{L}^{A}$	$p_{\mathrm{SC}}^{A}$	$p_{W}^{A}$
0.1	0.5	0.3	0.5	0.5	0.1668	0.1656	0.1769	0.1466	0.1618	0.1341	0.1523	0.1383	0.1565	0.1620	0.1278	0.2409
	0.6				0.2400	0.2374	0.2520	0.2156	0.2318	0.1894	0.2208	0.2032	0.2263	0.2333	0.1832	0.3249
	0.7				0.3394	0.3341	0.3526	0.3110	0.3254	0.2629	0.3151	0.2937	0.3217	0.3341	0.2607	0.4357
	0.8				0.4720	0.4612	0.4844	0.4408	0.4469	0.3585	0.4431	0.4169	0.4493	0.4729	0.3662	0.5766
	0.9				0.6459	0.6253	0.6541	0.6146	0.6008	0.4816	0.6152	0.5807	0.6167	0.6598	0.5064	0.7498
0.3	0.6				0.1310	0.1310	0.1429	0.1112	0.1202	0.0850	0.1141	0.1038	0.1207	0.1373	0.0892	0.1970
	0.7				0.2027	0.2001	0.2161	0.1776	0.1819	0.1292	0.1799	0.1627	0.1879	0.2067	0.1309	0.2800
	0.8				0.3110	0.3030	0.3242	0.2806	0.2722	0.1946	0.2821	0.2528	0.2899	0.3144	0.1931	0.4037
	0.9				0.4707	0.4519	0.4792	0.4366	0.4001	0.2890	0.4374	0.3868	0.4402	0.4767	0.2831	0.5823
0.5	0.8				0.1636	0.1564	0.1692	0.1421	0.1228	0.0764	0.1440	0.1208	0.1462	0.1712	0.0853	0.2283
	0.9				0.2690	0.2518	0.2703	0.2378	0.1958	0.1233	0.2406	0.1987	0.2400	0.2631	0.1266	0.3448
0.1	0.5	0.5	0.4	0.3	0.1399	0.1501	0.1629	0.1143	0.1382	0.1110	0.1236	0.1209	0.1335	0.1576	0.1252	0.2352
	0.6				0.2089	0.2231	0.2406	0.1782	0.2056	0.1668	0.1875	0.1845	0.2013	0.2282	0.1806	0.3183
	0.7				0.3068	0.3248	0.3471	0.2718	0.2992	0.2462	0.2803	0.2763	0.2978	0.3288	0.2588	0.4292
	0.8				0.4428	0.4619	0.4868	0.4049	0.4249	0.3558	0.4125	0.4056	0.4312	0.4686	0.3665	0.5713
	0.9				0.6287	0.6403	0.6624	0.5899	0.5886	0.5042	0.5980	0.5836	0.6109	0.6583	0.5107	0.7468
0.3	0.6				0.1097	0.1194	0.1309	0.0927	0.1015	0.0747	0.0967	0.0943	0.1049	0.1337	0.0889	0.1911
	0.7				0.1768	0.1882	0.2036	0.1533	0.1594	0.1194	0.1585	0.1535	0.1682	0.2027	0.1314	0.2729
	0.8				0.2845	0.2947	0.3137	0.2517	0.2481	0.1895	0.2593	0.2485	0.2685	0.3113	0.1954	0.3966
	0.9				0.4527	0.4532	0.4734	0.4062	0.3795	0.2959	0.4189	0.3961	0.4218	0.4771	0.2891	0.5771
0.5	0.8				0.1542	0.1532	0.1633	0.1268	0.1094	0.0732	0.1348	0.1227	0.1344	0.1709	0.0872	0.2225
	0.9				0.2642	0.2540	0.2668	0.2182	0.1804	0.1232	0.2326	0.2092	0.2269	0.2660	0.1307	0.3400
0.1	0.5	0.6	0.4	0.3	0.1478	0.1552	0.1678	0.1210	0.1453	0.1163	0.1307	0.1255	0.1391	0.1616	0.1292	0.2398
	0.6				0.2184	0.2283	0.2453	0.1865	0.2141	0.1721	0.1961	0.1894	0.2076	0.2330	0.1853	0.3234
	0.7				0.3170	0.3294	0.3504	0.2813	0.3085	0.2500	0.2896	0.2807	0.3041	0.3342	0.2642	0.4344
	0.8				0.4517	0.4645	0.4876	0.4150	0.4338	0.3560	0.4212	0.4078	0.4364	0.4740	0.3722	0.5760
	0.9				0.6325	0.6398	0.6602	0.5992	0.5950	0.4975	0.6040	0.5808	0.6130	0.6624	0.5161	0.7501
0.3	0.6				0.1140	0.1225	0.1337	0.0971	0.1065	0.0771	0.1008	0.0969	0.1079	0.1358	0.0914	0.1931
	0.7				0.1816	0.1918	0.2066	0.1595	0.1659	0.1217	0.1639	0.1562	0.1718	0.2057	0.1347	0.2757
	0.8				0.2884	0.2982	0.3161	0.2598	0.2562	0.1906	0.2654	0.2502	0.2719	0.3151	0.1998	0.4000
	0.9				0.4527	0.4555	0.4741	0.4160	0.3885	0.2940	0.4245	0.3948	0.4236	0.4817	0.2947	0.5806
0.5	0.8				0.1541	0.1556	0.1648	0.1313	0.1142	0.0739	0.1374	0.1234	0.1349	0.1737	0.0893	0.2241
	0.9				0.2618	0.2568	0.2681	0.2248	0.1873	0.1235	0.2360	0.2089	0.2266	0.2704	0.1335	0.3431
